# Electrothermally tunable cholesteric liquid crystal laser achieving 130 nm range with high circular polarization purity (|g| ≥ 1.4)

**DOI:** 10.1038/s41598-025-34461-x

**Published:** 2026-01-06

**Authors:** Mi-Yun Jeong, Keumcheol Kwak

**Affiliations:** 1 Department of Physics and Research Institute of Natural Science, Gyeongsang National University, Jinju, Republic of Korea; 2 Albatrace Inc., #813, 40 Omokcheon-ro 152 beon-gil, Gwonseon-gu, Suwon, Republic of Korea

**Keywords:** Cholesteric liquid crystal laser (CLC laser), Circularly polarized light (CPL), Electrothermal pitch tuning, Circular polarization dissymmetry factor (g-factor), Stokes-Mueller polarization analysis, Broadband tunable photonic bandgap, Optics and photonics, Physics

## Abstract

Circularly polarized light lasers are attracting growing attention for quantum optics, spin-optoelectronics, and next-generation display technologies. However, despite many demonstrations of cholesteric liquid crystal (CLC) lasers, two challenges remain: achieving broadband and continuous wavelength tunability and quantitatively determining the dissymmetry factor (g) under realistic conditions. Here, we present an actively tunable cholesteric laser based on supersaturated CLC (SCLC) cells driven by electrothermal control. In parallel SCLC cells, lasing wavelengths can be tuned about 130 nm (553–682 nm), but the spectral shift occurs discontinuously due to boundary constraints. In contrast, wedge-shaped SCLC cells form an electrothermally induced pitch gradient that enables rapid and continuous spectral tuning across ~ 100–126 nm. To rigorously quantify the polarization state, we employed the Stokes–Mueller analysis with three circular analyzers, correcting for non-ideal transmission and leakage. Across the full 130-nm tuning range, the generated laser maintained a consistently high degree of circular polarization purity, with the dissymmetry factor g quantified as 1.633 at 600–620 nm and 1.40 at 630–650 nm ranges. These results verify that the circular polarization performance of the SCLC-based tunable laser remains robust throughout the entire visible lasing spectrum. Differential scanning calorimetry further revealed second-order SmA–CLC–isotropic phase transitions, clarifying dynamic asymmetries between heating and cooling and guiding optimal voltage-sweep strategies. Together, these findings demonstrate a reliable pathway toward broadband, color-tunable, and spin-selective CLC lasers, bridging photonic bandgap engineering with practical applications in nanophotonics, spin-optoelectronics, and advanced display technologies.

**Supplementary Information:**

The online version contains supplementary material available at 10.1038/s41598-025-34461-x.

## Introduction

Circularly polarized lasers are emerging as key elements enabling next-generation photon technologies where deterministic spin control of photons is essential^1,2^. Target applications span spin-based optoelectronic engineering^3^, quantum information processing^4,5^, three-dimensional displays^6^, and polarization-encoded optical communications^7^, all of which offer the benefits of their ability to generate and route photons with a set manual operation and high polarization purity^1,2^. Among the various circularly polarized laser sources, cholesteric liquid crystals (CLCs) are occupying a unique region in continuously tuneable lasing in the full visible spectrum range^8,9^. Their self-assembled helical structures form one-dimensional chiral photonic crystals representing circular Bragg reflection bands, or photonic band gap (PBG), reflecting one handedness while transmitting the opposite one^10–12^.

In this work, we demonstrate an electrothermally tunable single-layer SCLC laser that achieves a wide tuning range of approximately 130 nm across the visible spectrum while simultaneously maintaining a high dissymmetry factor in the generated circularly polarized laser emission. In CLCs, the PBG enables mirrorless microcavity lasing, with emission occurring near the band edges at n_o_$\:\times\:$P and n_e_$\:\times\:$P, where P is the helical pitch, which is the periodic helical structure length, and n_o_ and n_e_ are the ordinary and extraordinary refractive indices of the CLC, respectively^8,9,13,14^. The PBG center wavelength λ_c_ is P$\:\times\:$(n_o_+n_e_)/2. By varying the pitch, the PBG can be effectively tuned^8,9^. However, as quantitative circularly polarized laser sources, two practical challenges have limited the use of CLC lasers. First, although many demonstrations focus on the possibility of wavelength tuning, polarization purity often remains uncharacterized, assuming ideal analyzer behavior. In practice, light passing through a circular analyzer (e.g., a CLC filter or a commercial circular polarizer) indicates abnormal transmission and leakage^15–18^. Second, in broadband across the visible spectrum, fast and reversible tuning is difficult to achieve while maintaining stable spin selectivity. Therefore, simultaneously addressing both issues of broad-spectrum agility and rigorous quantification of polarization purity is an important step towards practical spin-selective light sources^19,20^.

Our prior studies have progressively advanced this agenda. In 2021, we reported a polymer-stabilized CLC (PCLC) wedge-cell that operated as a multifunctional optical component circular polarizer, notch filter, band pass filter and beam splitter exhibiting ultrahigh circularly polarized light purity (|g| ≈ 1.9, the PCLC wedge cell is not for laser but filter) and long-term thermo-optical stability^20^. And we have achieved a fast and continuous lasing with supersaturated CLC (SCLC) wedge cells, based on electrothermal effects that form pitch gradient arrays within 10 min, to implement dynamically tunable lasers in full visible spectral range^9^. As another example of generating high-quality circular polarization light, Yuyang Pu et al. recently reported circular polarization with an asymmetric coefficient of up to 1.80 using a composite film of up-conversion nanoparticles (UCNPs) and perovskite nanocrystals (PNCs) integrated with a cholesteric liquid crystal (CLC) polymer film. But in this research, they generated only a few discrete wavelengths^21^.

Further from the recent development of continuous wavelength-tuned laser systems covering the full visible spectrum^9^, in this work we showed the high quality circularly polarized laser light from the SCLC system. The quality of circularly polarized light is confirmed by the degree of circular polarization coefficient (g) compensating non-ideal transmission condition. Conventional characterization with a linear polarizer and a wavelength-matched quarter-wave plate evaluates only a single wavelength at a time and therefore cannot verify polarization purity throughout a continuously tunable lasing bandwidth. To overcome this limitation, we adopt a measurement strategy that enables broadband determination of the degree of circular polarization across multiple lasing wavelengths. Our goal is to rigorously investigate and verify the polarization purity of these generated laser beams, increasing their applicability and importance as a reliable light source for pure circular polarization in various optoelectronic and spin-photon applications.

In experiment, using an incomplete analyzer (almost all circular polarization filters deviate from the ideal value of 50% for reflectance and transmittance), we find that the actual “how to easily determine quantitative and reproducible values when determining the degree of circular polarization (the asymmetric coefficient, g) of a CLC laser beam” is needed. Thus, a combination of Stokes-Mueller format and three circular analyzers was used to calibrate leakage and non-ideal permeability to obtain a reliable lower limit, with the dissymmetry factor g quantified as 1.633 at 600–620 nm and 1.40 at 630–650 nm ranges confirming that broadband wavelength agility and strong circular polarization can be achieved simultaneously within a single SCLC platform. Furthermore, from the study of differential scanning calorimetry (DSC) to clarify the thermodynamic path (SmA–CLC–isotropy) and the dynamic asymmetry between heating and cooling, we have found out the thermo-electric property of the SCLC cells and an optimal high-voltage to low-voltage sweep strategy for maximizing pitch tuning bandwidth. Together, these elements connect photon bandgap engineering with polarization measurements to establish CLC lasers as reliable and integrable CPL sources over a wide spectrum without the need for sophisticated nanoproduction^22–24^ for quantum photonics, spin optics, and advanced display systems. We believe that these strategies allow for practical advances in the field through CPL lasers whose wavelength agility is consistent with quantitatively validated circular polarization purity.

## Results and discussion

### Fabrication of SCLC-laser cells and CLC filters

Two types of CLC cells were fabricated for laser generation experiments: One is a parallel P-SCLC1 cell with a uniform thickness (1.5 cm x 1.5 cm x 25 μm) and the other types were wedge shaped CLC cells with W-SCLC2 cells (1.5 cm x 4.5 × (20 μm, 40 μm)) and W-SCLC3 cells (1.3 cm x 4.0 cm x (15 μm, 20 μm)) constructed to have thickness gradients by placing thin and thick spaces at 1.8 cm intervals. To apply voltage in these CLC cells, we employed an ITO substrate as a transparent electrode with a surface resistivity of 15 Ω/sq. On the ITO surfaces, as an alignment layer, a polyimide (PI, 7492 K, Nissan Chemical Korea Co. Ltd., Korea) layer was spin coated and crosslinked at 170 °C. And then PI layer was rubbed with a rubbing cloth (HC20, cotton, NESTECHNOLOGY Co., Ltd, Korea). The three empty cells of P-SCLC1 cell, W-SCLC2 cell, and W-SCLC3 cell were filled with a supersaturated CLC (SCLC) by a capillary method, respectively. The SCLC is composed of a nematic liquid crystal (MLC6608, Merck Korea) with a negative dielectric constant, a chiral molecule (R811, 45 wt%, Merck Korea), and with two laser dyes (DCM [4-dicyanomethylene-2-methyl-6-p-dimethylaminostyryl-4Hpyran] and LDS698, both in ~ 0.5 wt%, Exciton). The solubility of R811 at 45 wt% concentrations in CLC varies greatly with temperature change and is in a supersaturated CLC (SCLC) state (see ref. 9). The SCLC exhibit highly thermosensitive PBG properties, with λ_c_ change rates up to > 100 nm/°C, see Fig. S1.

Three types of filters have been prepared to measure the degree of circular polarization of the generated laser pulses from the P-SCLC1 and the W-SCLC2 cells. One is a commercially available circular polarizer, F-88,100 (#88–100, Edmund Optics Co., Polarizing Efficiency, *P* > 99.98%) that is composed of a linear polarizer and a quarter wave plate and allows only left circularly polarized light to pass through in the spectrum range of 400 nm to 700nm^25^.

And the other four filters made in our lab are UV cured polymerized CLCs, a F-LCLC613, a F-RCLC613, a F-LCLC648, and a F-RCLC648. The F-LCLC613 and F-LCLC648 have left-handed helicity and the F-RCLC613 and F-RCLC648 have right-handed helicity: as a substrate to make the CLC filters, BK7 plates were used, one side surface of the each BK7 substrate was coated with an antireflection layer (AR layer, spectrum range from 400 nm to 1000 nm, thickness 0.6 μm, Yunam Optics, Korea)^20^. The AR layer can improve refractive index mismatch between the air and the BK7 layer of the CLC cells. And the other side of the each BK7 surface was spin-coated with the polyimide (PI, 7492 K) layer and then thermally cross-linked. Each PI layer was also rubbed with a rubbing cloth (HC20). Empty cells with ~ 15 μm thickness were fabricated from two substrates consisting of PI/BK7/AR layers with PI coated surface facing inward.

As a cholesteric liquid crystal for the F-RCLC613 filter fabrication, solvent removed ~ 0.2 g of RMS11-066 (with λ_B_ = ~ 400 nm, Merck) and ~ 0.21 g of RM141C (nematic LC, Merck) were mixed. And for the F-RCLC648 filter fabrication, solvent removed ~ 0.2 g of RMS11-062 (with λ_B_ = ~ 650 nm, Merck). And for a F-LCLC613 cell filter fabrication, solvent removed 0.2 g of RM-xx (with λ_B_ = ~ 400 nm) and 0.19 g of RM141C were mixed. And for a F-LCLC648 filter, solvent removed 0.2 g of RM-xx (with λ_B_ = ~ 400 nm) and ~ 0.15 g of RM141C were mixed. The RMS11-066, RMS11-062, and RM-xx are solutions of photo reactive mesogens in toluene initially, so, the solvent in the solutions were evaporated completely before use. And each of the four RM-CLC solutions were filled in a previously made empty cell at about 70 °C. Subsequently, the four CLC cells were polymerized by exposure to a UV lamp (25 mW/cm^2^ at 365 nm) for about 30 min. Therefore, we fabricated the F-RCLC613 with λ_B_ = ~ 615 nm and the F-LCLC613 with λ_B_ = ~ 613 nm, the F-RCLC648 with λ_B_ = ~ 648 nm, and the F-LCLC648 with λ_B_ = ~ 643 nm.

**Fig. 1:**
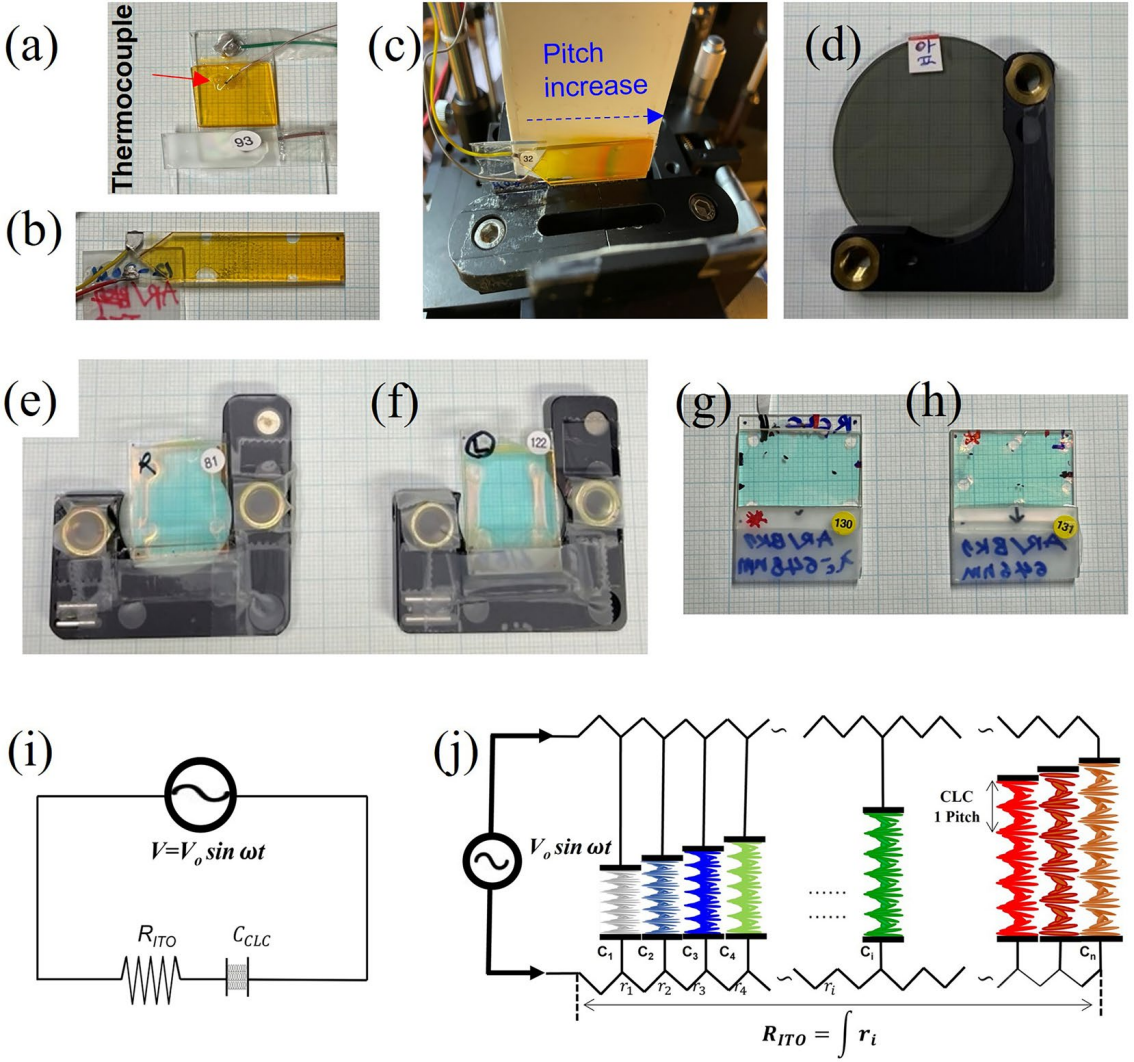
Photos of the fabricated SCLC cells and filters: (**a**) the parallel P-SCLC1 cell, (**b**) the wedge W-SCLC2 cell, (**c**) the wedge W-SCLC2 cell with a pitch gradient under applied voltage, the color change formed in the horizontal direction (wedge direction) of the cell can be visually confirmed that the pitch gradient is formed, (**d**) the F-88,100 cell, (e) the F-RCLC613 cell, (**f**) the F-LCLC613 cell, (**g**) the F-RCLC648 cell, (**h**) the F-LCLC648 and (**i**) a circuit diagram of the parallel SCLC cell structure, and (**j**) a schematic diagram of the wedge SCLC3 cell shown in (**c**); V, input AC voltage; R_ITO_, the resistance of the employed ITO substrate; $\:{C}_{CLC}$, capacitance of SCLC1 cell.

Figure 1 shows photos of the fabricated two SCLC cells for laser generation and five filters: (a) the dye-doped parallel P-SCLC1 cell, (b) the dye-doped wedge W-SCLC2 cell, (c) the dye-doped wedge W-SCLC2 cell with a pitch gradient under applied voltage, see the Fig. S2 for more detailed wedge cell structure, (d) the filter F-88,100 cell, (e) the CLC filter F-RCLC613 cell, (f) the CLC filter F-LCLC613 cell, (g) the CLC filter F-RCLC648 cell, (h) the CLC filter F-LCLC648 cell, and (i) a circuit diagram of the parallel SCLC cell structure, and (j) a schematic diagram of the wedge SCLC2 cell shown in (c).

### Principle of pitch change in the SCLC cells

Meanwhile, as shown in the Fig. 1f, the SCLC1 cell forms an $\:{R}_{ITO}$-$\:{C}_{cell}\:$series circuit^9^, where $\:{R}_{ITO}$ is a resistance of the ITO layer, $\:{C}_{ITO}=\:\frac{{\epsilon\:}_{0}{\epsilon\:}_{s}A}{d}\:$ is a capacitance of the SCLC layer, and$\:\:{\epsilon\:}_{0},\:{\epsilon\:}_{s},\mathrm{A},\:\mathrm{a}\mathrm{n}\mathrm{d}\:\mathrm{d}\:$are the vacuum permittivity, the dielectric permittivity of the SCLC at the low frequency limit, the electrode area, and the distance between electrodes, respectively^9^. When a voltage is applied to the $\:{R}_{ITO}$-$\:{C}_{cell}\:$circuit of the cell, at a specific frequency $(f_{{PR}} = 1/(2\pi \:R_{{ITO}} C_{{cell}} ))$, dielectric heating^26–28^ can occur depending on the applied voltage due to pseudo-dielectric relaxation^28–30^. Through the dielectric heating at the SCLC cell, the pitch could be changed and the PBG could move to a short wavelength.

The W-SCLC2 cell in Fig. 1(c and j) forms an array with continuous many $\:\int\:\left({{r}_{i},c}_{i}\right)$ circuits connected along the wedge direction. The intensity of the voltage decreases with increasing thickness d in the SCLC2 wedge cell. Thus, in wedge SCLC cells with high-sensitivity PBG properties, the temperature decreases with increasing thickness, forming continuous pitch gradient arrays along the wedge direction at once^9^.

### Optical properties of the CLC filters

**Fig. 2:**
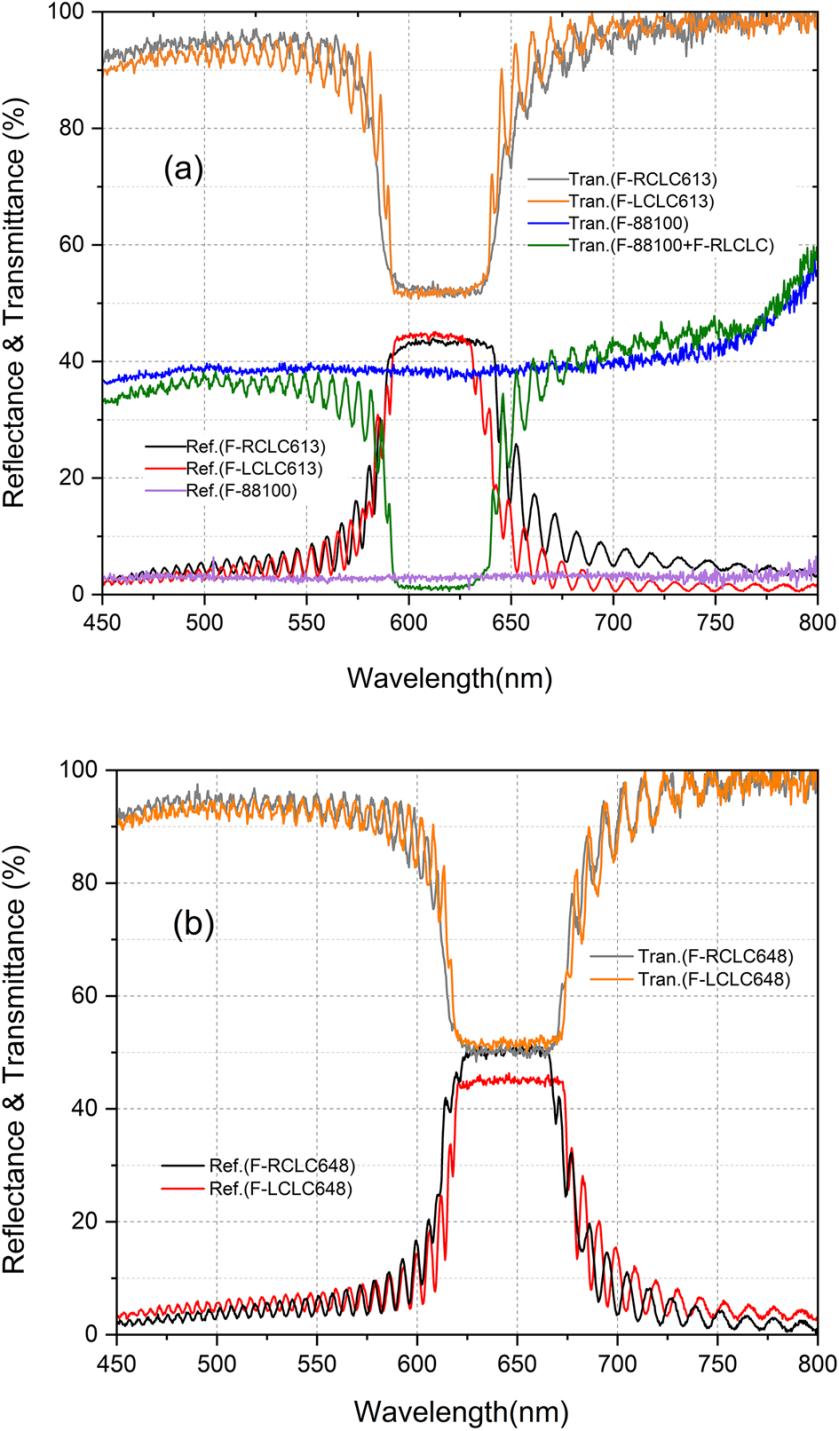
Reflectance and transmittance of the circular-polarizer-filters: (**a**) reflectance of (F-RCLC613 cell, ─), (F-LCLC613 cell, ), and (F-88100, ), transmittance of (F-RCLC613 cell, ), (F-LCLC613 cell, ), (F-88100, ), and (F-88100 and F-LCLC, ), respectively. (**b**) reflectance of (F-RCLC648 cell, ─), (F-LCLC648 cell, ), transmittance of (F-RCLC648 cell, ), and (F-LCLC648 cell, ), respectively.

Figure 2 (a) and (b) show the reflectance(Ref.) and transmittance(Tran.) spectra of the five circular-polarizer filters. In the Fig. 2 (a); reflectance of (F-RCLC613 cell, 43.4% in the PBG, ─), (F-LCLC613 cell, 44.5% in the PBG, ), and (F-88100, 2.8%, ), transmittance of (F-RCLC613 cell, 52% in the PBG, ), (F-LCLC613 cell, 52% in the PBG, ), (F-88100, 38.7%, ), and (F-88100 and F-LCLC, ), respectively and in the Fig. 2 (b); reflectance of (F-RCLC648 cell, 50% in the PBG, ─), (F-LCLC648 cell, 45% in the PBG, ), transmittance of (F-RCLC648 cell, 50% in the PBG, ), and (F-LCLC cell, 51% in the PBG, ), respectively. For the F-RCLC613 and F-RCLC648 which reflect right circular polarized light in the PBG and F-LCLC613 and F-LCLC648 which reflect left circular polarized light in the PBG, respectively. Although the transmittance and reflectance were improved by the AR coating layer between the air and the BK7 layer, it was slightly out of the ideal 50%. Because the CLC filters are composed of multilayer films, air/glass/ITO/PI/SCLC/PI/ITO/glass/air film, it is thought that the refractive index mismatch between two adjacent layers is the cause.

In Fig. 2, the transmittance by using both F-88,100 and F-LCLC613 () is 1.1% within the PBG of the F-LCLC613: when the initial unpolarized light passes through F-88,100, it has a left circularly polarized state, and when this light passes through F-LCLC613 again, the transmittance value is expected to be 0%, because the F-LCLC613 reflects only the left circularly polarized light in the PBG. However, this 1.1% transmittance corresponds to 2.8% ((1.1/38.8) x 100%) of the transmittance of F-LCLC613 due to the reflectance of not 50% but 44.5% () of the above F-LCLC613. Values where the reflectance and transmittance of these filters deviate from 50% can lead to an error of up to ~ 13%.

### Dynamic properties of the molecules in the SCLC by DSC

Previously, using the electric-thermal effect using the SCLC, we achieved very good results in implementing continuous wavelength tunable laser generation over the full visible spectrum range in just 10 min, but the supersaturated CLC (SCLC) samples tended to show different dynamic properties depending on the experimental conditions (e.g., ambient temperature, heat treatment conditions before first applying voltage to the SCLC cell, etc.). So, detailed differential scanning calorimeter (DSC, TA Instrument-Waters Ltd.) measurements were performed to find conditions to always reproduce the same PBG and laser wavelength changes over a wide wavelength range and to determine the order of the SmA-CLC phase transition.

**Fig. 3:**
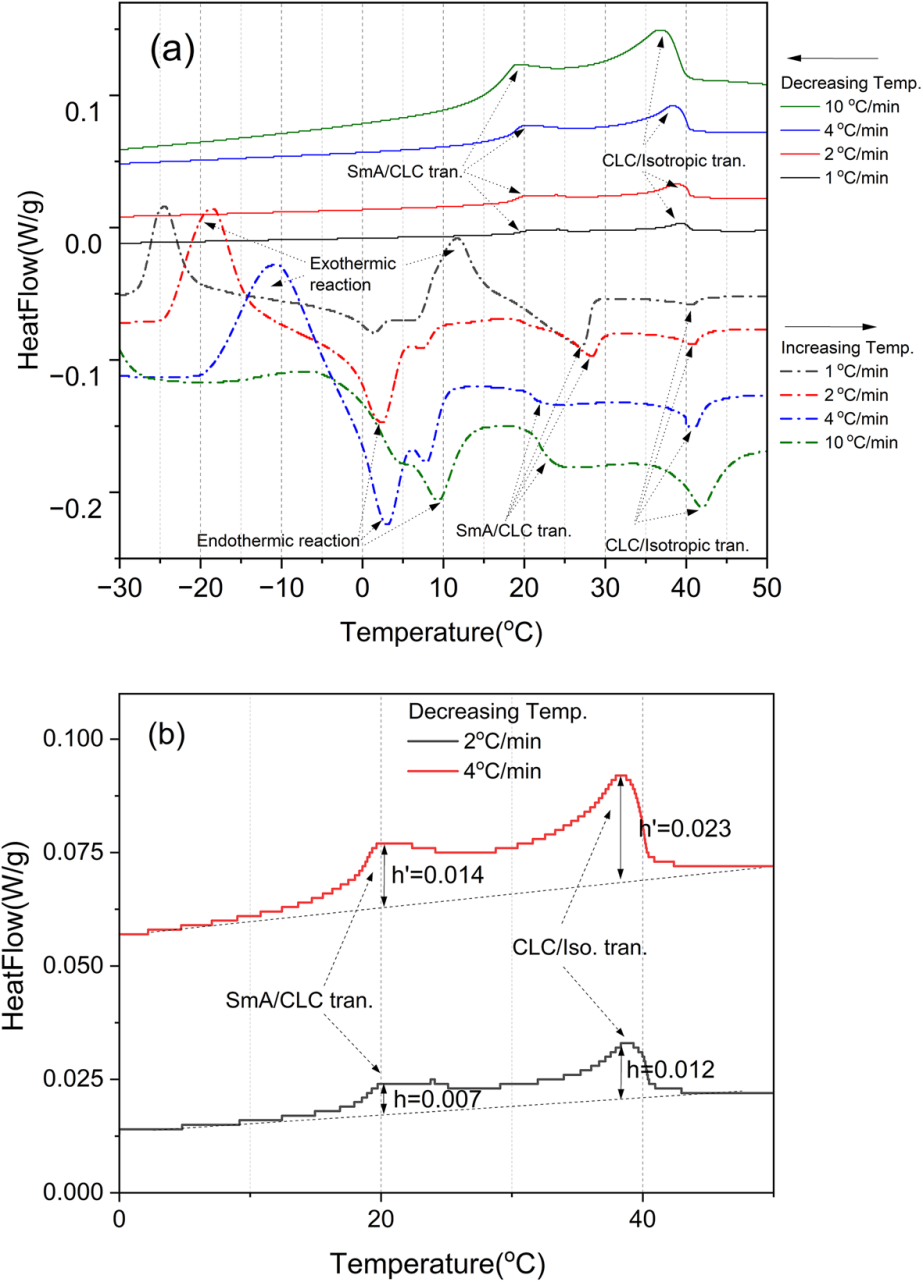
DSC traces of the SCLC sample. (**a**) DSC traces of the SCLC sample, at 4 different decreasing temperature rates of 1-, 2-, 4- and 10 °C/min with increasing and then decreasing the temperature, respectively. (**b**) N (2), $\:h$ and $\:{h}^{{\prime\:}}$ values of the SCLC sample from the DSC traces.

In DSC, experiments were carried out sequentially at four different heating rates using one sample: 1 °C/min, 2 °C/min, 4 °C/min, and 10 °C/min, increasing the temperature and then decreasing the temperature, Fig. 3 DSC (a). The measurement results unexpectedly show very different dynamic behavior between the process of increasing and decreasing the temperature. In the increasing temperature process, the temperature of exothermic and endothermic reactions moved to higher temperatures as the heating rate increased from 1 °C/min to 10 °C/min and the temperature range representing the CLC phase is increased. The endothermic transition appears to superimpose the two peaks, indicating that the SCLC is composed of a mixture. But interestingly, for the decreasing temperature process, the dynamic behavior of the four different heating rates was almost same, the temperature range of CLC phase is almost same and does not show any exothermic and endothermic reactions. This important result show that when voltage is applied to the SCLC cell to cause a pitch change, the measurement must be made by starting the experiment at a high voltage and then reducing the voltage applied to the SCLC cell to widen the pitch-changing wavelength range and this result was applied to laser generation experiments using SCLC. To rule out damage caused by repeated DSC measurements (four runs for one sample), DSC was repeated for four fresh SCLC samples over four heating rates and the same results were obtained. There is a well-known DSC technology that distinguishes phase transitions of liquid crystals from primary and secondary^31–33^. It is based on the measure of the ratio N = $\:h$/$\:{h}^{{\prime\:}}$, where $\:h$ is the height of the transition peak recorded for a mass of sample m and a heating rate $\:{\dot{T}}_{p}$ and $\:{h}^{{\prime\:}}\:$is the height of the transition peak recorded for the same mass of sample m and at twice heating rate, $\:2{\dot{\:T}}_{p}$. From the measured ratio (N) of the peak heights of the DSC trace, when it is isothermal first order phase transition, N will have the value, 1 < N<$\:\sqrt{2}$, and in the cases of 1st order phase transition with impure materials or the 2nd order phase transition, N will have value near 2^31–33^. Therefore, from the measured DSC trace data of SCLC sample in the Fig. 3(b) at the heating rate 2 °C/min and 4 °C/min, the N values of them are obtained as ~ 2. So, for both (Isotropic→CLC phase, at 38.27 °C) and (CLC phase→SmA phase, at 20.28 °C) transitions are the 2nd order phase transitions. This result can be confirmed again in the results of the dynamic characteristics experiment of the following CLC molecules (see Fig. 6).

### Active wavelength tuning of laser emission and photonic band gap

The laser generation experiment was conducted using the P-SCLC1 and the W-SCLC2 (Fig. 1. (a and b)), and the advantages and disadvantages of the two cells were compared. To maximize the spectral range of lasing wavelength through CLC pitch change, the DSC experimental result of the previous section was applied: first, a high voltage was applied to the SCLC cell, and then the laser wavelength change was measured by lowering the voltage. See Fig. S3 for an experimental setup to measure PBG changes and laser generation in the P-SCLC1 and the W-SCLC2 cells and a description of the process of investigating the degree of circular polarization of laser pulses produced in these cells.

**Fig. 4:**
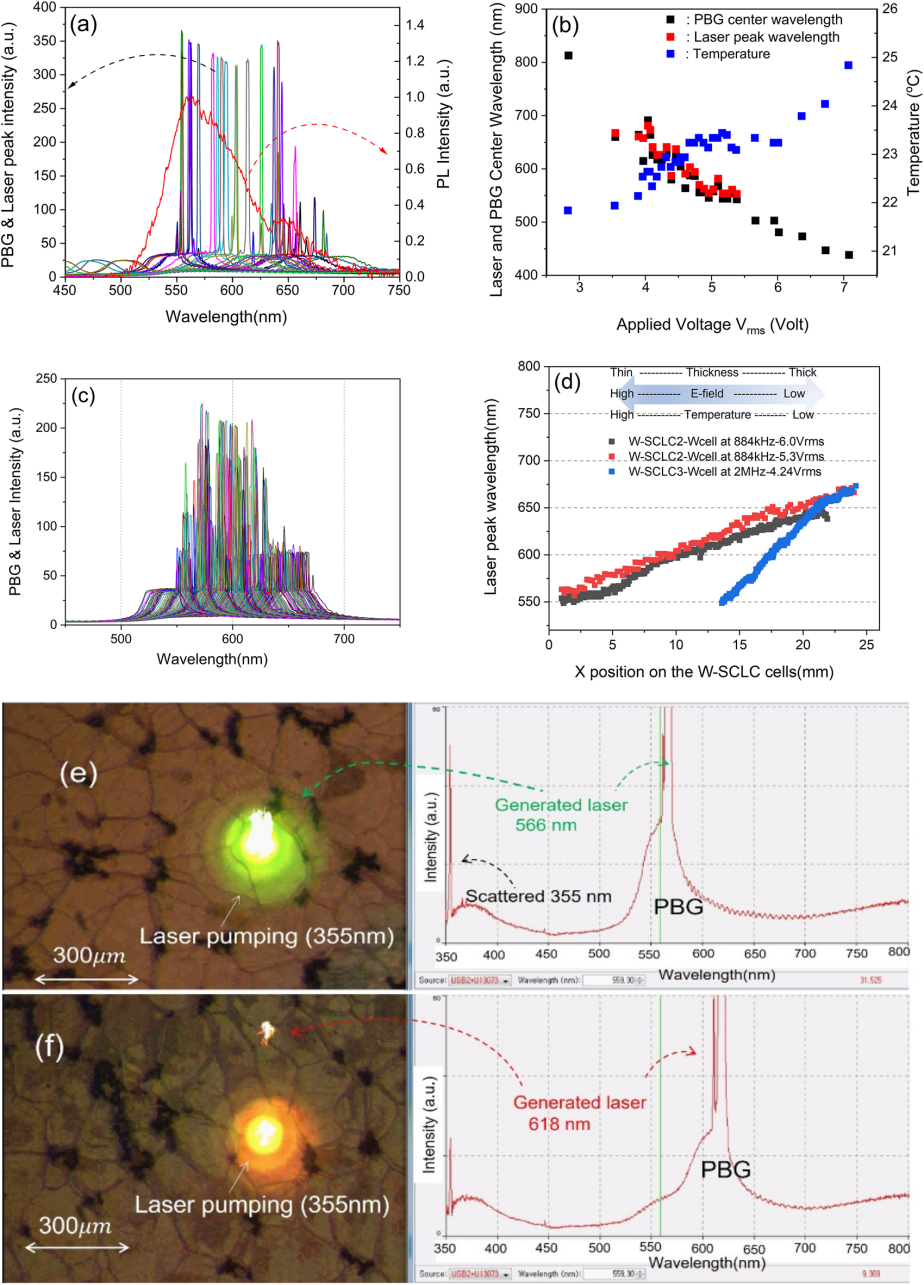
(**a**) The generated laser lines and photonic band gaps by changing AC voltage at 3 MHz and PL spectrum of the P-SCLC1 cell. (**b**) The change of laser peak wavelength (red filled squares), PBG center wavelength (black filled squares), and temperature change (blue filled squares) shown in (**a**). (**c**) Generated laser spectra as a function of spatial x-position from the W-SCLC3 cell with 4.24$\:\:{\mathrm{V}}_{\mathrm{r}\mathrm{m}\mathrm{s}}$ at 2.0 MHz. (**d**) The change of laser peak wavelength position as a function of spatial x-position from the W-SCLC2 cell of with 6.0$\:\:{\mathrm{V}}_{\mathrm{r}\mathrm{m}\mathrm{s}}$ at 884 kHz (black filled squares) and with 5.3$\:\:{\mathrm{V}}_{\mathrm{r}\mathrm{m}\mathrm{s}}$ at 884 kHz (red filled squares), and from the W-SCLC3 cell with 4.24$\:\:{\mathrm{V}}_{\mathrm{r}\mathrm{m}\mathrm{s}}$ at 2.0 MHz (blue filled squares), respectively. (**e**) and (**f**) CCD photos of lasing pulses of 566 nm and 618 nm on the surface of the P-SCLC1 cell and their spectra of PBG and laser pulses at the lasing moment, respectively. (**e**) and (**f**) show the laser images and spectra of the PBGs and laser peaks at 566 and 618 nm in the texture of the W-SCLC2 cell, respectively.

Figure 4(a) shows generated laser lines from the parallel P-SCLC1 cell by applying external voltages, at room temperature, $\:\sim22\pm\:0.3\:$^o^C. In the entire laser generation experiment, the temperature remained within 22 ± 0.3 °C during the tuning measurements using a thermoelectrically stabilized sample holder to remove the thermal drift associated with the high thermal sensitivity (> 100 nm/°C) of the SCLC. For broadband tuning, we used an AC-driven frequency of 3 MHz, as higher driving frequencies enable uniform pitch modulation at lower applied voltages. On the other hand, larger voltages were used to investigate field-dependent pitch responses at low frequencies of 884 kHz. That is, as the applied voltage increases, the frequency decreases, and as the voltage decreases, the frequency must increase^9,29^.

**Fig. 5:**
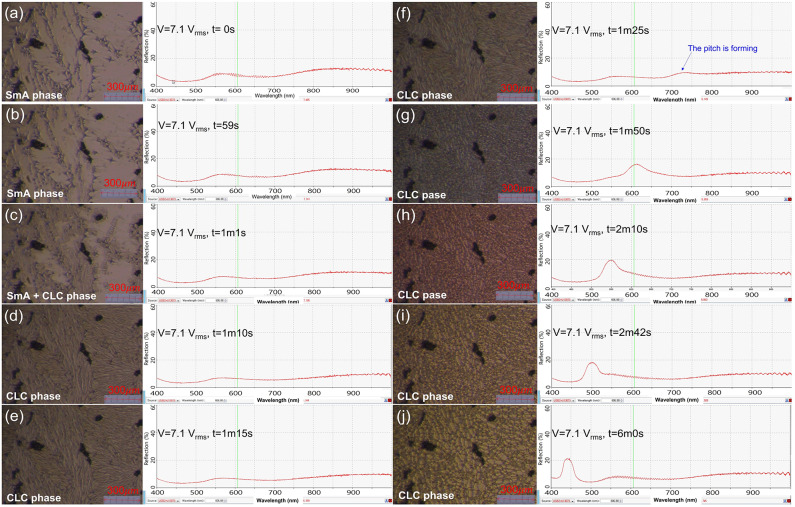
Photos of the P-SCLC1 cell texture and spectra by the CCD and the spectrophotometer under in-situ measurements of phase transition from a SmA phase to the CLC phase of the cell by applying a voltage of AC 7.07 V_rms_ at 4 MHz.

We can see a photo luminescence (PL) spectrum (red line) and generated laser peaks pumped by the YAG laser at the 355 nm, and PBG spectra of the P-SCLC1 cell. After applying AC voltage, 7.07$$\:{\:V}_{rms}$$ to the P-SCLC1 cell at 3 MHz, we gradually reduced the applied voltage while maintaining the frequency: the $$\:{\varDelta\:V}_{rms}$$= ~ 0.7 V in the PBG-only moving section and the $$\:{\varDelta\:V}_{rms}$$= ~ 0.07 V in the laser-generated region. Each voltage was held for ~ 10 min. to ensure that the CLC pitch change in the cell reached a fully stable point. The generated laser wavelength gradually changed from 553 nm at 5.37 $$\:{\:V}_{rms}$$ to 682 nm at 4.03 $$\:{\:V}_{rms}$$. It changed discontinuously $$\:\varDelta\:\lambda\:=4\sim9\:nm$$. This discontinuous laser tuning is caused by the boundary condition (BC) in the parallel CLC cell with uniform thickness. Helical pitch of the CLC cell with an alignment layer should be quantized with the number of half-pitch to satisfy the BC, or $$\:\left(n+1\right)\cdot\:\left(\raisebox{1ex}{${P}_{1}$}\!\left/\:\!\raisebox{-1ex}{$2$}\right.\right)=n\cdot\:\left(\raisebox{1ex}{${P}_{2}$}\!\left/\:\!\raisebox{-1ex}{$2$}\right.\right)$$, where P_1_ and P_2_ are adjacent pitches with $$\:{P}_{1}<{P}_{2}:$$ the characteristics of pitch in the CLC cell is well explained in ref.^34,35^. It can be seen that the laser pick occurs discontinuously even though the applied voltage is changed very small, $$\:{\varDelta\:V}_{rms}$$= ~ 0.07 V, and thick thickness ~25$$\:\:\mu\:$$m of cell due to the boundary conditions of the cell. Figure 4(b) shows, for the P-SCLC1 cell, the laser peak wavelength (red filled squares), the PBG center wavelength (black filled squares), and the surface temperature change (blue filled squares) under dielectric heating of the $$\:{R}_{ITO}$$-$$\:{C}_{cell}$$ circuit. Figure 4(c) shows laser line spectra as a function of x-position (wedge direction) of the W-SCLC3 cell at 4.24$$\:\:{\mathrm{V}}_{\mathrm{r}\mathrm{m}\mathrm{s}}$$ and 2.0 MHz. As the pump beam moves along the wedge direction, the lasing wavelength changes continuously, indicating the pitch gradient was formed by a temperature gradient. Figure 4(d) summarizes the change of laser peak wavelength as a function of spatial x-position. For the W-SCLC2 cell with spaces (20 $$\:\mu\:m,\:40$$ $$\:\mu\:m):$$ at 5.3$$\:\:{\mathrm{V}}_{\mathrm{r}\mathrm{m}\mathrm{s}}$$ and 884 kHz (red filled squares) the emission tunes from 555.8 nm to 671.0 nm ($$\:\varDelta\:\lambda\:=116.2\:\mathrm{n}\mathrm{m}\:$$) over 23.2 mm with $$\:\varDelta\:\mathrm{x}=200{\upmu\:}\mathrm{m};\:$$ at 6.0$$\:\:{\mathrm{V}}_{\mathrm{r}\mathrm{m}\mathrm{s}}$$ and 884 kHz (black filled squares) it tunes from 548.6 nm to 646.9 nm ($$\:\varDelta\:\lambda\:=99.3\mathrm{n}\mathrm{m}\:$$) over 20.9 mm with $$\:\varDelta\:\mathrm{x}=50{\upmu\:}\mathrm{m}$$, respectively. For the W-SCLC3 cell with spaces (12 $$\:\mu\:m,\:20$$ $$\:\mu\:m)$$ at 4.24$$\:\:{\mathrm{V}}_{\mathrm{r}\mathrm{m}\mathrm{s}}$$ and 2 MHz (blue filled squares), the laser tuned from 548.8 nm to 673.3 nm ($$\:\varDelta\:\lambda\:=125.5\:\mathrm{n}\mathrm{m}$$) over 10.95 mm with $$\:\varDelta\:\mathrm{x}=50\:{\upmu\:}\mathrm{m}$$. We could see that by adjusting the amplitude of the applied voltage and the cell thickness, the spectral range of lasing could be controlled actively. The laser data used in Fig. 5 (c and d) were reconstructed from^9^.

Comparing the rate of laser wavelength change in parallel and wedge cells, approximately 10 min after the change of the applied voltage is required for pitch change to change from one wavelength to another in parallel cells. On the other hand, in wedge cells, a spatial pitch gradient is formed within about 10 min of a single fixed voltage being applied, creating hundreds of continuously varying pitch arrays simultaneously. To change the wavelength, only the pump’s position needs to be moved in the wedge direction. Depending on the experimental needs, these individual operations in parallel cells can still be useful. We also find that the wedge cells exhibit much more continuous wavelength changes than the parallel cells, even in the PBG changes. And the laser peak intensity of P-SCLC1 cell (25 μm thick) is more than 50% higher than that of W-SCLC3 cell (12 μm to 20 μm thick). This is mainly because P-SCLC1 cell was thicker to secure relatively more laser gain, the second reason was that constant intensity of pump beam was used for W-SCLC3 cell, whereas for P-SCLC1 cell, the intensity of pump beam was relatively high over short and long wavelength range to obtain larger laser peak.

Figure 4(e) and (f) show the laser images and spectra of the PBGs and laser peaks at 566 and 618 nm in the texture of the W-SCLC2 cell, respectively. They were acquired simultaneously in situ by means of a CCD camera and a spectrophotometer (OOI).

### In-situ study on the SmA-CLC phase transition using CCD

Next, to study the dynamics of molecules and phase transitions more directly, the room temperature was lowered to ~ 20 ± 0.3 °C, just below (near) the SmA-CLC phase transition temperature.

Figure 5 shows 10 photographs of field measurements of the phase transition and pitch change from SmA to CLC phase by applying a voltage of AC 7.07 V_rms_ at 4 MHz. For measurements, the following results were measured using the previously used P-SCLC1 cells. More high frequency (or high voltage) was required to overcome the low temperature of the cell because the room temperature was 2 °C lower than in the case of the Fig. 4(a) measurement. The Fig. 5(a) shows the texture of SmA before applying voltage to the cell. After applying the voltage, when ~ 1 min has passed, a change of molecular alignment appeared in Fig. 5(c) by the electrothermal effect. It can be seen from Fig. 5(c) to (f) that molecular rearrangement is occurring gradually for 30 to 40 s of changing from SmA to CLC phase, and finally, the CLC phase appears. And as the next step, the CLC pitch continuously decreases and the domain size can be expanded simultaneously (see Fig. 5(c) to (j), video data V1). Real-time dynamic research on P-SCLC1 cells confirms that the phase change progressed gradually, and this result is the same as the DSC experiment result, indicating that the phase change from SmA to CLC is secondary.

Figure 5 mainly describes the SmA–CLC phase transition process and the resulting electrothermal pitch evolution, but it also confirms that the formation of the photonic band-gap during field-induced pitch modulation does not degrade optical coherence. Because the SCLC employs a nematic liquid crystal with negative dielectric anisotropy, the applied electric field preserves the helical lattice rather than disrupting it during tuning. After the phase transition, the emerging PBGs maintain their bandwidth, reflectance contrast, and spectral sharpness throughout the tuning process, consistent with the sustained narrowband lasing over the ~ 130 nm tuning range shown in Fig. 4.

### The degree of circular polarization g of the generated laser light

From the results of the laser generation experiment in the previous section, it can be seen that up to 130 laser wavelengths can be generated in a single SCLC cell, and quantitatively determining the degree of circular polarization g of the laser light generated from this SCLC cell is very important in future applications of these lasers. In this work, the circular dissymmetry factor g is defined as, $\:g\equiv\:{2(S}_{3}\:/\:{S}_{0}\:)=2({I}_{L}\:-\:{I}_{R})/\:({I}_{L}+\:{I}_{R}),\:$ where $\:{S}_{0}(=$ $\:{I}_{L}+\:{I}_{R})\:and$ $\:{S}_{3}(={I}_{L}\:-\:{I}_{R}$) are Stokes parameters^36^. Here, $\:{I}_{L\:}\mathrm{a}\mathrm{n}\mathrm{d}\:\:{I}_{R}$ denote the laser intensities obtained using circular-polarization–selective filters that preferentially transmit the left-handed and right-handed circular polarization dominant components, respectively, rather than providing ideal full separation. Therefore, the degree of circular polarization of the laser peaks generated in the SCLC cells were investigated.

**Fig. 6:**
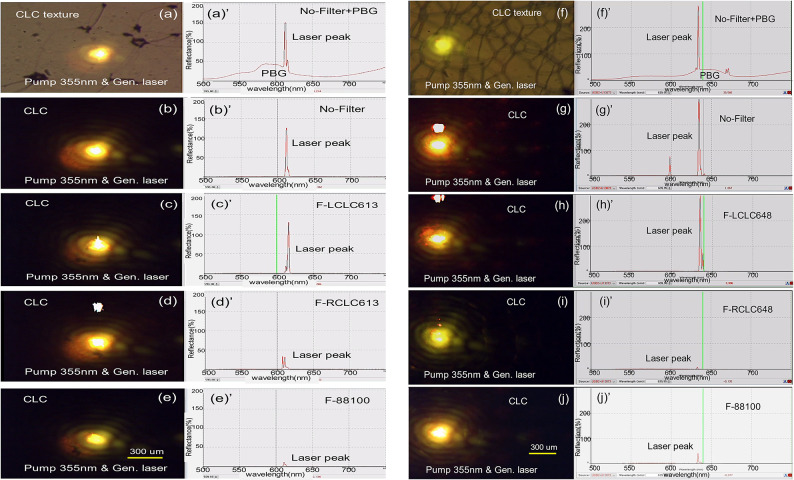
CCD images of laser pulses generated at the surface of the W-SCLC2 cell (**a**–**e**) and their corresponding laser intensities: without any circular polarizer filter (**a’**, **b’**), after passing through F-LCLC (**c’**), F-RCLC (**d’**), and F-88,100 (**e’**) in the 600–620 nm range, respectively. CCD images of laser pulses (**f**–**j**) and their corresponding laser intensities: without any circular polarizer filter (**f’**, **g’**), after passing through F-LCLC (**h’**), F-RCLC (**i’**), and F-88,100 (**j’**) in the 630–650 nm range, respectively.

For the two different wavelength regions where the laser is generated in the W-SCLC2 cell with right-hand helicity (Fig. 1(b) and (c)), Fig. 6 (a-e) in the 600-620-nm spectrum range and Fig. 6 (f-j) in the 630-650-nm range show the change in laser intensity after passing through each of the five previously prepared circular polarization filters, F-88,100, F-RCLC613, F-LC613, F-RCLC648, and F-LC648, respectively. The reason why the two different wavelength regions were handled separately is that the quantum efficiency of the laser dyes according to the change in wavelength is different (see the PL spectrum in Fig. 4(a)), and the width of the optical band gap of the CLC filters used in the analysis is narrow to cover both areas. Using the respective filters in the two spectral ranges, (600 nm to 620 nm) and (630 nm to 650 nm), the largest laser beam was selected and the average value was used to calculate the dissymmetry factor g value. This is also to avoid laser picks with weakened laser intensity when the pump laser beam encounters defects (aggregated dye lumps) present in the SCLC cell during laser generation. Video data related to Fig. 6 has been added as supporting information (V2,V3,V4,V5, and V6).

The CCD photos of laser generation (Fig. 6(a ~ j) confirm that the laser pulses we selected as data are well generated on the W-SCLC2 cell surface.

In Fig. 6, panels (i) and (i’) are simultaneous measurements of the same laser pulses generated in W-SCLC2 cells, respectively: (i) is a CCD image of the cell surface and (i’) is a spectrum collected through an optical waveguide (see Figure S3). To measure the laser pick and PBG simultaneously, the panels (a and a’) and (f and f’) recorded the PBG and laser simultaneously by irradiating the pumper beam with the OOI white light source irradiated on the W-SCLC2 surface. The CCD image (a and f) shows the laser occurrence at the point where the pumping laser is incident on the W-SCLC2 texture, and the spectrum (a’ and f’) shows the laser occurrence at the PBG edge. However, when the lamp is turned on, the reflected “L3” light from the CCD zoom lens surface (see Fig.S3) enters the waveguide, distorting the PBG and laser peak intensity as shown in (a’ and f’). To address this issue, the rest of the data measured the panel (b-e and g-j) and (b-e and g-j)’ with the OOI lamp turned off, measuring only the intensity of the pure laser light. Panel (b’ and g’) shows the laser intensity spectrum without an analyzer at the waveguide input. Panel (c’ and h’) shows the laser intensity with the F-LCLC613 cell and F-LCLC648 cells at the waveguide input, respectively; because F-LCLC613/-648 reflects left circular polarized light and thus transmits right circular polarized light, the laser intensity is largely preserved—consistent with right circular polarized light emission from the right-handed W-SCLC2 cell. In contrast, with F-RCLC613/-648 (panel (d’ and i’), reflects right circular polarized light) and with the commercial F-88,100 (panel (e’ and j’), left circular polarized light -pass), the laser intensity is strongly attenuated. These trends indicate predominantly right circular polarized laser light emission from the W-SCLC2 cell. For a quantitative estimate, five pulses within (600–620 nm) and (630–650 nm) under each analyzer condition (see Figure S4) were averaged (see Fig. S5) to compute the dissymmetry factor g, see the Videos V2–V6.

In the case of the laser generated by the P-SCLC1 parallel cell, similar results were obtained using the F-LCLC613 and F-RLCLC613 cells, Fig. S6.

To quantitatively verify that the circular polarization purity is preserved over the full tunable range, we compared five polarization-selective conditions at two spectrally separated lasing bands centered near 610 nm and 640 nm.

**Fig. 7:**
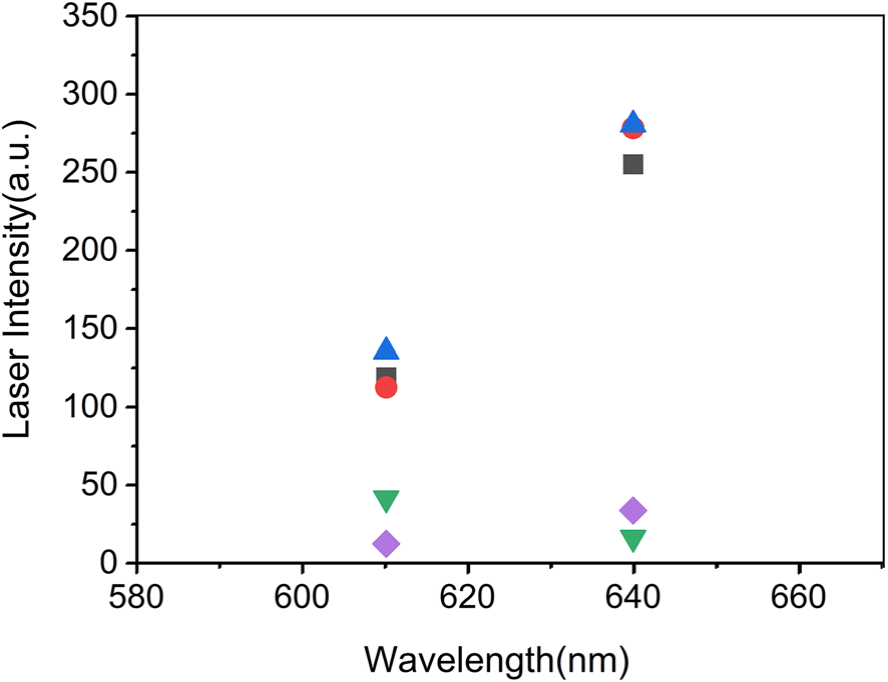
Wideband circularly polarized purity of the laser produced in the W-SCLC2 cell. In two spectral separation laser bands centered near 610 nm and 640 nm, the average laser intensity is shown for five polarization selection measurement conditions (No-filter + PBG (black filled squares), No-filter (red filled circles), F-LCLC613/F-LCLC648 (blue filled triangles), F-RCLC613/F-RCLC648 (green filled inverted triangles), and F-88100 (purple filled diamonds)). In both bands, the handy matching filters (F-LC613 and F-LC648) have high transmission intensity, whereas the handy miss matching filters (F-RCLC613, F-RCLC648, and F-88100) strongly suppress the transmission intensity. On the other hand, the case of No-Filter and No-Filter + PBG indicates the initial laser intensity S_0_ generated in the W-SCLC2 cell. The higher absolute intensity observed at 640 nm was due to the use of a strong pump beam in the 630–650 nm region. It can be seen that the relative polarization selective contrast remains unchanged in both wavelength regions. The more pronounced separation at 640 nm is thought to be due to the improved circular selection performance of F-RCLC648 and F-LCLC648 filters, which is closer to a 50% design that is more ideal than F-RCLC613. The results show that high circular polarization purity will be maintained throughout the approximately 130 nm tuning range.

Figure 7 summarizes the mean laser intensities measured with No-filter + PBG, No-filter, F-LCLC613, F-RCLC613, F-LCLC648, F-RCLC648, and F-88,100 for both wavelength intervals. The absolute intensities at 640 nm are slightly higher because a stronger pump beam was used to reach the lasing threshold in the 630–650 nm interval; this change in pump level affects the overall signal magnitude but does not alter the relative circular-polarization contrast. In addition, the circular analyzers fabricated for the 648 nm region (F-RCLC648 and F-LCLC648) are closer to the ideal 50% circular-selective reflectance/transmittance design than the earlier F-RCLC613 and F-LCLC613 filters, resulting in a more clearly resolved separation between the handedness-matched (No-filter + PBG, No-filter and F-LCLC648) and mismatched ( F-RCLC648, and F-88100) conditions at 640 nm. The consistent contrast pattern observed at both wavelengths confirms that the dissymmetry factor and circular polarization purity remain robust across the ~ 130 nm tuning range of the SCLC laser.

Unlike polarization characterization with a linear polarizer and a wavelength-matched quarter-wave plate, which evaluates only a single wavelength at a time and tends to overestimate circular polarization due to non-ideal analyzer leakage, the approach used here allows broadband and reproducible determination of the dissymmetry factor g across multiple lasing wavelengths. Several CLC-based circular analyzers with partially overlapping photonic band-gaps cover different regions of the visible spectrum, and each analyzer simultaneously probes multiple wavelengths within its band while its non-ideal transmission properties are explicitly calibrated within the Stokes–Mueller framework. This enables the rigorous verification of high circular polarization purity over the full ~ 130-nm tuning range, rather than numerical refinement of a single wavelength.

### Calculation of the circular polarization dissymmetry factor g  

In this section, we derived the expression for the dissymmetry factor (g) and calculated the (g) value of laser peaks generated by the W-SCLC2 cell under realistic (non-ideal) analyzer performance. The three circular polarization filters (F-RCLC613, F-LCLC613, and F-88100) were employed in the 600–620 nm spectrum range and the three circular polarization filters (F-RCLC648, F-LCLC648, and F-88100) were employed in the 630–650 nm spectrum range, respectively. For the g value calculation, the measured experimental data (Figs. 2 and 6, Figure S4, and Table-S1, Table-S2) were used.

Let the generated laser peak intensity be $\:I={I}_{L}+\:{I}_{R}$, where $\:{I}_{L}$ and $\:{I}_{R}$ are the left- and right-circular components, respectively. The Stokes parameters are $\:{S}_{0}=$ $\:{I}_{L}+\:{I}_{R}\:$, $\:{S}_{3}={I}_{L}\:-\:{I}_{R}$^36^. In circularly polarized luminescence(CPL), the dissymmetry factor is defined as

1 $$\:g\equiv\:{2(S}_{3}\:/\:{S}_{0}\:)=2({I}_{L}\:-\:{I}_{R})/\:({I}_{L}+\:{I}_{R}),\:\:-2\le\:g\le\:2.$$

For a non-ideal circular analyzer with transmission coefficients $\:{\tau\:}_{L}\:and\:{\tau\:}_{R}$ for left circular polarized light and right circular polarized light, the transmitted intensity is^37^, see [S-P1]

2 $$\:I=\frac{1}{2}\:\:\left[\left({\tau\:}_{L}+{\tau\:}_{R}\right){S}_{0}+\left({\tau\:}_{L}-{\tau\:}_{R}\right){S}_{3}\right]=\:{\tau\:}_{L}{I}_{L}+{\tau\:}_{R}{I}_{R}\:$$

For an unpolarized incident light, the average transmittance $\:{T}_{UP}=\:\frac{\left({\tau\:}_{L}+{\tau\:}_{R}\right)}{2}$.

For F-LCLC613 and F-RCLC613, define $\:A\equiv\:\:{\tau\:}_{pref\:}\left(\mathrm{t}\mathrm{r}\mathrm{a}\mathrm{n}\mathrm{s}\mathrm{m}\mathrm{i}\mathrm{s}\mathrm{s}\mathrm{i}\mathrm{o}\mathrm{n}\:\mathrm{o}\mathrm{f}\:\mathrm{t}\mathrm{h}\mathrm{e}\:\mathrm{p}\mathrm{r}\mathrm{e}\mathrm{f}\mathrm{e}\mathrm{r}\mathrm{r}\mathrm{e}\mathrm{d}\:\mathrm{h}\mathrm{e}\mathrm{l}\mathrm{i}\mathrm{c}\mathrm{i}\mathrm{t}\mathrm{y}\right)\:and\:B\equiv\:\:{\tau\:}_{leak\:}\left(\mathrm{t}\mathrm{r}\mathrm{a}\mathrm{n}\mathrm{s}\mathrm{m}\mathrm{i}\mathrm{s}\mathrm{s}\mathrm{i}\mathrm{o}\mathrm{n}\:\mathrm{o}\mathrm{f}\:\mathrm{t}\mathrm{h}\mathrm{e}\:\mathrm{o}\mathrm{p}\mathrm{p}\mathrm{o}\mathrm{s}\mathrm{i}\mathrm{t}\mathrm{e}\:\mathrm{h}\mathrm{e}\mathrm{l}\mathrm{i}\mathrm{c}\mathrm{i}\mathrm{t}\mathrm{y}\right),$ Then $\:A+B=2\cdot\:{T}_{UP}=1.04\:$ (since $\:{T}_{LCLC}{= 52\%, \:T}_{RCLC}=52\%$, see Fig. 2 (or Table S1). Because real analyzers are imperfect, A ≤ 1 and B > 0.

F-LCLC613 preferentially transmits right circular polarized light (leaking left polarized light) and F-RCLC613 preferentially transmits left polarized light (leaking right circular polarized light).

When $\:{I}_{LCLC}\:$is laser intensity after passing through F-LCLC613 and, $\:{I}_{RCLC}\:$is laser intensity after passing through F-RCLC613, hence, see [S-P**2**, S-P3]

3 $$\:{I}_{LCLC}=A\cdot\:{I}_{R}+B\cdot\:{I}_{L},$$

4 $$\:{I}_{RCLC}=A\cdot\:{I}_{L}+B\cdot\:{I}_{R}$$

Summing Eqs. (3) and (4) gives $\:{I}_{LCLC}+\:{I}_{RCLC}=\left(A+B\right)\cdot\:\left({I}_{L}+{I}_{R}\right)=1.04\cdot\:{S}_{0}.$

Using the measured values (Table S1 and Table S2) $\:{I}_{LCLC}=$ 134.9 and $\:{I}_{RCLC}$= 41.1,

5 $$\:{S}_{0}=({I}_{LCLC}+\:{I}_{RCLC})/\left(A+B\right)=\:\frac{134.9+41.1}{1.04}=169.23$$

Taking difference,

6 $$\:{I}_{LCLC}-\:{I}_{RCLC}=-\left(A-B\right)\cdot\:\left({I}_{L}-{I}_{R}\right)=-\left(A-B\right){S}_{3}$$

For the commercial circular polarizer (F-88100), let $\:{t}_{L}\:and\:{t}_{R}$ denote its left /right circular polarization transmission coefficients^38^. With unpolarized transmittance $\:{T}_{UP,88100}=0.3868,$ We have $\:{t}_{L}+\:{t}_{R}=$2$\:\cdot\:{T}_{UP,88100}=0.7736.$ The measured transmitted intensity is I_88100_ =12.02, so

7 $$\begin{gathered} I_{{88100}} = \frac{1}{2}\left[ {\left( {t_{L} + t_{R} } \right)S_{0} + \left( {t_{L} - t_{R} } \right)S_{3} } \right] \hfill \\ 2I_{{88100}} - \left( {t_{L} + t_{R} } \right)S_{0} = \left( {t_{L} - t_{R} } \right)\left( {I_{L} - I_{R} } \right) \hfill \\ \end{gathered}$$

By combination of (6) and (7),

8 $$\begin{aligned} & \frac{{\left( {t_{L} - t_{R} } \right)}}{{\tau _{{pref}} - \tau _{{leak}} }} = \frac{{\left( {t_{L} + t_{R} } \right)S_{0} - 2I_{{88100}} }}{{I_{{LCLC}} - I_{{RCLC}} }} \\ & = (0.7736*169.23 - 2*12.02)/(134.9 - 41.1) \approx 1.13984 \\ \end{aligned}$$

With $$0 \le \:t_{{L,\:R}} \le \:1,\:\left| {t_{L} - t_{R} } \right|\: \le \:0.7736.$$ Therefore,

9 $$\:\left|A-B\right|\:\le\:\:\frac{\:\left|{t}_{L}-{t}_{R}\right|}{1.13984}\:\le\:\:\frac{0.7736}{1.13984}\:\approx\:0.679$$

Finally, $$\:g = \frac{{2\left( {I_{L} \: - \:I_{R} } \right)}}{{S_{0} }} = \frac{{2\left| {I_{{LCLC}} - \:I_{{RCLC}} } \right|}}{{S_{0} \left| {\tau \:_{{pref}} - \tau \:_{{leak}} } \right|}}\: \ge \:\left( {\frac{{2 \times \:93.8}}{{169.23 \times \:0.679\:}}} \right) \approx \:1.633\:,$$ where 93.8 = 134.9–41.1.

In the case of the 630 nm to 650 nm spectrum range using F-LC648 and F-RCLC648, and F-88,100, the g value was calculated in the **S-Cal** of the Supporting Information, and a rather small value was calculated with a value of g ≥ 1.4. It is thought that some large values of the laser pick are saturated beyond the y-axis limit value, and the intensity of the laser pick is reduced when the No filter and F-LC648 filters are used.

Thus, explicitly accounting for non-ideal diattenuation with three circular analyzers yields a rigorous lower bound g ≥ 1.4 for the SCLC laser, confirming strongly right-circularly polarized emission.

## Conclusion

We have realized a cholesteric liquid crystal (CLC) system that can generate lasers with a high degree of circular polarization while being able to tune wavelengths in the broadband spectrum region using electro-thermal effects. Applying the results of electrothermal dynamic characteristics of DSC and the SCLC cells, we achieved continuous laser tuning of approximately 100–126 nm in wedge-shaped SCLC cells, while parallel cells were found to be wider but discontinuous laser wavelength tuning of approximately 130 nm due to boundary condition. To confirm the quality of the degree of circular polarization of the generated laser, we used three circular analyzers within the Stokes-Mueller framework to correct the leakage in the non-ideal analyzer and set a reliable lower limit of g = 1.633 at 600–620 nm and 1.40 at 630–650 nm ranges. Importantly, by quantifying the dissymmetry factor g, this establishes SCLC lasers as a practical broadband circular polarization laser source capable of maintaining strong spin selectivity without spectral trade-off. These results demonstrate a robust path to compact, spin-selective, tunable CLC lasers that combine quantitative polarization verification with practical spectral control. Continuously tunable lasers in spatially high-resolution wedge cells and stepwise tuning in parallel cells can provide modes that complement spin-photonics, polarized multiplex displays, and quantum photonic devices. DSC results also noted that starting with a high voltage and decreasing to a low voltage broadens the laser tuning range in the SCLC cell and that both the SmA→CLC and Iso→CLC phases are secondary. Finally, from the results of this paper, it is carefully expected that the laser beams generated from the previously helical nanostructured CLC resonator cell will all have a high circular polarization and can be applied to CLC laser beams in the ultraviolet and infrared spectrum ranges as well as the entire visible light area, so further verification will be carried out in the future.

## Experimental method

### Lasing and PBG measurement by applying an AC voltage

In the laser experiments, AC voltages were applied to the P-SCLC1 and W-SCLC2,-3 cells using a function generator (DAGATRON FG-8210, 10 MHz, Korea). Laser action in the SCLC cells was pumped by a 355 nm Q-switched Nd: YAG laser (7 ns, 10 Hz). To avoid damaging the SCLC cell, the pump pulse energy was limited to ≤ 4 µJ, and a fused-silica lens (f = 20 cm) focused the ~ 2 mm-diameter beam onto the SCLC cell surface. The beam waist w at the cell surface was approximately 71 μm, estimated from w ≈ λ/θ (where λ is the pump wavelength and θ is the beam divergence). The photonic band gap (PBG) and laser spectra, together with the cell-surface temperature, were monitored simultaneously using a spectrophotometer (HR2000+, Ocean Optics (OOI), USA), a surface-mounted thermocouple( Lake Shore, TC-E-36-03, USA) on the P-SCLC1 cell, and a CCD camera with a zoom lens (AC1300-30gc, Basler, Germany).

## Supplementary Information

Below is the link to the electronic supplementary material.

Supplementary Material 1

Supplementary Material 2

Supplementary Material 3

Supplementary Material 4

Supplementary Material 5

Supplementary Material 6

Supplementary Material 7

## Data Availability

Data supporting the results of this study are available in the supplemental material in this article.

## References

[1] Suárez-Forero, D. G., Jalali Mehrabad, M., Vega, C., González-Tudela, A. & Hafezi, M. Chiral quantum optics: recent developments and future directions. *PRX Quantum*. **6**, 020101. 10.1103/PRXQuantum.6.020101 (2025). APS Journals.

[2] Maksimov, A. A. et al. Circularly polarized laser emission from an electrically pumped chiral microcavity. *Phys. Rev. Appl.* **17**, L021001. 10.1103/PhysRevApplied.17.L021001 (2022). Physical Review.

[3] Li Wan, R. et al. Sensitive near-infrared circularly polarized light detection using chiroptically active organic photodiodes. *Nat. Photonics* 17, 649–656 (2023). 10.1038/s41566-023-01230-z. Nature.

[4] Lee, S. J. et al. Ki Tae Nam, and Hong-Gyu Park, Spin angular momentum–encoded single-photon emitters in WSe₂ coupled with chiral plasmons. *Sci. Adv.* 10, eadn7210 (2024). 10.1126/sciadv.adn7210. Science. 10.1126/sciadv.adn7210 PMC11122662 38787944

[5] Mujie Rao, J. et al. Chiral single photon routing via Cavity-Assisted Spin-Momentum locking. *Nano Lett.* **25**, 11406–11412. 10.1021/acs.nanolett.5c02598 (2025). PubMed. 40622228 10.1021/acs.nanolett.5c02598

[6] Zhang, M. et al. Taotao Zhuang, and Shu-Hong Yu, processable circularly polarized luminescence materials for flexible 3D displays. *Sci. Adv.* **9**, eadi9944. 10.1126/sciadv.adi9944 (2023). SciencePMC. 37878702 10.1126/sciadv.adi9944 PMC10599622

[7] Miao, W. C. et al. Metasurface-driven polarization-division multiplexing of PCSEL for optical communications. *eLight* 3, 50 (2023). 10.1186/s43593-023-00065-6. PubMedPMC. 10.1186/s11671-023-03935-0 PMC10704011 38062340

[8] Mi-Yun Jeong; Jeong Weon Wu, continuous Spatial tuning of laser emissions in a full visible spectral range. *International J. Mol. Sciences* **12** : 2007–2018. (2011). 10.3390/ijms12032007. PMC. 10.3390/ijms12032007 PMC3111647 21673936

[9] Mi-Yun Jeong; Hyeon-Jong Choi; Youngwoo Nam; keumcheol Kwak, quickly formed continuously tuneable laser in the full visible spectrum using cholesteric liquid crystal cells based on the electrothermal effect. *Laser & Photonics Reviews* (2024). 10.1002/lpor.202301256

[10] Muhammad, F. Akhlesh Lakhtakia, The circular Bragg phenomenon. *Advances in Optics and Photonics* 6 : 225–292. (2014). 10.1364/AOP.6.000225. Optica Publishing Group.

[11] , W. D. St. John,, W. J. Fritz,, Z. J. Lu &, D. K. Yang Bragg reflection from cholesteric liquid crystals. *Phys. Rev. E*. **51**, 1191–1198. 10.1103/PhysRevE.51.1191 (1995). 10.1103/physreve.51.1191 9962762

[12] Pochi, Y. *Claire Gu, Optics of Liquid Crystal Displays* 2nd edn (Wiley, 2009). (in Chap. 7 Optical Properties of Cholesteric Liquid Crystals).

[13] De Gennes, P. G. & Prost, J. *The Physics of Liquid Crystals* 2nd edn (Clarendon, 1993).

[14] Ding, Y. et al. Polarization conversion effect in cholesteric liquid crystal-based polarization volume gratings. *Opt. Express*. **32**, 25, 44425 (2024).

[15] Ding, Y. et al. Broadband and wide-view cholesteric liquid crystal polarization selective reflectors. *Opt. Express*. **33** (15), 30967–30976. 10.1364/OE.569217 (2025). 40733884 10.1364/OE.569217

[16] Shirvani-Mahdavi, H., Mohajerani, E. & Wu, S. T. Circularly polarized high-efficiency cholesteric liquid crystal lasers with a tunable nematic phase retarder. *Opt. Express*. 18(5) :5021-7. (2010). 10.1364/OE.18.005021. PMID: 20389514. 10.1364/OE.18.005021 20389514

[17] Zhang, J., Zhang, Y., Yang, J. & Wang, X. Beyond color boundaries: pioneering developments in cholesteric liquid crystal photonic actuators. *Micromachines* **15** (6), 808 (2024). 38930778 10.3390/mi15060808 PMC11205596

[18] Lotfi Saadaoui, D. et al. Zenghua Gan, Yigang Li and Jingjun Xu, electrically tunable Two-Color cholesteric laser. *Polymers* **15** (24), 4656 (2023). 38139908 10.3390/polym15244656 PMC10747753

[19] Zhang, J., Zhang, Y., Yang, J. & Wang, X. Beyond color boundaries: pioneering developments in cholesteric liquid. *Cryst. Photonic Actuators Micromachines*, **15**(6), (2024). 10.3390/mi15060808 PMC11205596 38930778

[20] Jeong, M. Y., Choi, H. J., Kwak, K. & Yu, Y. Multifunctional optical device with a continuous tunability over 500 Nm spectral range using polymerized cholesteric liquid crystals. *Polymers* **13**, 3720 (2021). 34771278 10.3390/polym13213720 PMC8587428

[21] Yuyang Pu, X. et al. Guofu Zhou, upconversion circularly polarized luminescence with dissymmetry factor up to 1.80 from flexible Perovskite-Liquid crystal membrane. *Chem. Eng. J.* **512**, 162515 (2025).

[22] Zhang, Y. H. et al. Zhi-Feng Zhang & Yan-Qing Lu, logical rotation of non-separable States via uniformly self-assembled chiral superstructures. *Nat. Commun.* **15**, 1108 (2024). 38321000 10.1038/s41467-024-45299-8 PMC10847456

[23] Gu, H. et al. Lakshminarayana Polavarapu, Guofu Zhou, and Xiao-Fang Jiang, Color-Tunable lead halide perovskite Single-Mode chiral microlasers with exceptionally high g_lum_. *Nano Lett.* **24**, 13333 (2024). 39361829 10.1021/acs.nanolett.4c03838 PMC11503764

[24] Jeong, M. Y., Chung, K. S. & Wu, J. W. Optical properties of laser lines and fluorescent spectrum in cholesteric liquid crystal laser. *J. Nanosci. Nanotechnol*. **15**, 10, 7632 (2015). 26726387 10.1166/jnn.2015.11176

[25] Edmund optics Co. data sheet of Circular polarizer, # 88–100.

[26] Wen, C. H. & Wu, S. T. Dielectric heating effects of dual-frequency liquid crystals. *Appl. Phys. Lett.* **86**, 231104 (2005).

[27] Yin, Y., Shiyanovskii, S. V. & Lavrentovich, O. D. Electric heating effects in nematic liquid crystals. *J. Appl. Phys.* **100**, 024906 (2006).

[28] Wu, P. C., Wu, G. W., Yu, C. H. & Lee, W. Voltage-induced pseudo-dielectric heating and its application for color tuning in a thermally sensitive cholesteric liquid crystal. *Liq Cryst.* **46**, 2085 (2019).

[29] Wu, P. C., Wu, G. W., Timofeev, I. V., Zyryanov, V. Y. & Lee, W. Electro-thermally tunable reflective colors in a self-organized cholesteric helical superstructure. *Photon Res.* **6**, 1094 (2018).

[30] Perkowski, P. et al. Technical aspects of dielectric spectroscopy measurements of liquid crystals. *Opto-Electron Rev.* **16**, 271 (2008).

[31] Navard, P. & Haudin, J. M. The height of DSC phase transition peaks I. Theory. *J. Therm. Anal.* **29**, P405 (1984).

[32] Navard, P. & Haudin, J. M. The height of DSC phase transition peaks: II. Some applications to liquid crystals. *J. Therm. Anal.* **29**, P415 (1984).

[33] COX, R. & P. NAVAKD and Study of the smectic a nematic transition in octyl and Nonyl cyanobiphenyl. *Mol. Cryst. Liq Cryst.* **102** (Letter,), 261–264 (1984).

[34] Jeong, M. Y. & Wu, J. W. Continuous Spatial tuning of laser emissions with tuning resolution less than 1 Nm in a wedge cell of dye-doped cholesteric liquid crystals. *Opt. Express*. **18**, 24221 (2010). 21164768 10.1364/OE.18.024221

[35] Jeong, M. Y. & Wu, J. W. Continuous Spatial Tuning of Laser Emissions in a Full Visible Spectral Range. *Int. J. Mol. Sci*., 12, (2011). (2007). 10.3390/ijms12032007 PMC3111647 21673936

[36] Trent, R. W., Dissertation, A., Azzam & Bashara, N. M. Ellipsometry and Polarized Light, Chs. 1–3, North-Holland, Chs. 1–3, (1977).

[37] Chipman, R. A., Lam, W. S. T. & Young, G. Polarized Light and Optical Systems, Chs. 4–6, CRC (2018/2020).

[38] Goldstein, D. H. Polarized Light, 3rd ed., Chs. 3–4, CRC Press (2011).

