# Supplementary material for: Electrothermally tunable cholesteric liquid crystal laser achieving 130 nm range with high circular polarization purity (|g| ≥ 1.4)
Source: Sci Rep. 2026 Jan 6;16:4281. doi: 10.1038/s41598-025-34461-x (PMC12859137; doi:10.1038/s41598-025-34461-x)
Supplement: Supplementary file 1 — Supplementary Material 1 [file 41598_2025_34461_MOESM1_ESM.pdf]

Electrothermally Tunable Cholesteric Liquid Crystal Laser Achieving 130 nm Range with High Circular Polarization Purity (g ≥1.4)

Mi-Yun Jeong[1,*] and Keumcheol Kwak[2]

[1] Department of Physics and Research Institute of Natural Science, Gyeongsang National University, Republic of Korea

[2] Albatrace Inc., #813, 40 Omokcheon-ro 152 beon-gil, Gwonseon-gu, Suwon, Republic of Korea.

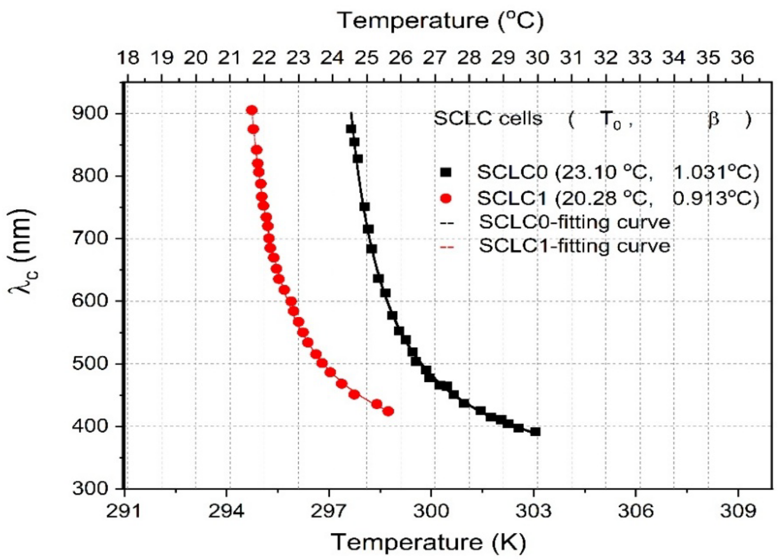

Fig. S1 λ_c change with temperature of the two CLC cells: the SCLC0 cell has no dye and the SCLC1 has two laser dyes of DCM and LDS698. They were fitted by the Keating theory ($\lambda_c(T) = \gamma({}^{T_0}/_{T})(1 + {}^{\beta}/_{(T - T_0)})^2$), with $R2 \geq 0.997$), where T_0 is the SmA - CLC phase transition temperature in Kelvin (K), γ and β are fitting parameters representing the sensitivity to the temperature change of the SCLC, γ depends on the anharmonicity factor, interplanar distance and molecular moment of inertia.

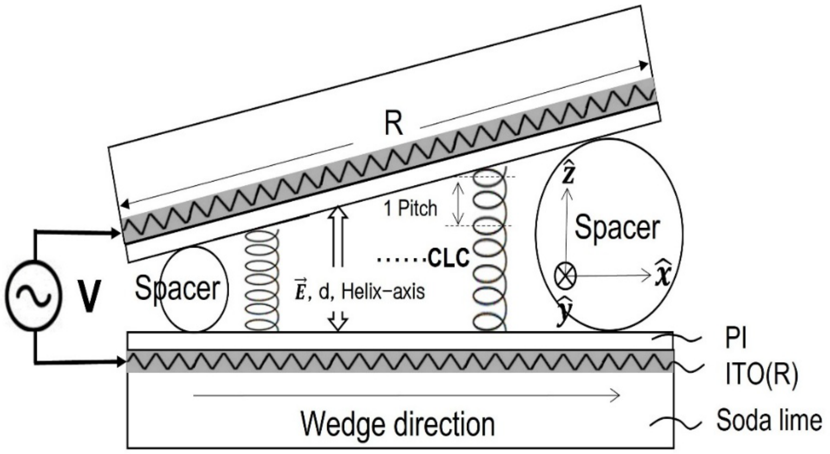

Fig. S2 Schematic diagram of the wedge cell structure with a continuous pitch gradient realized through the electrothermal effect: V, applied AC voltage; R, total resistance of the ITO substrate; PI, polyimide layer; ITO, indium tin oxide; soda lime, glass substrate; $\vec{E}$, electric field; d, thickness at a position of the cell.

Supplementary Video data:

Video V1. Real-time phase transition from the SmA to the CLC phase)

Real-time video of the phase transition from the SmA to the CLC phase. The transition starts at 1 min 1 s and lasts for approximately 25 s, followed by a decrease in the CLC pitch toward shorter wavelengths, corresponding to the data shown in Figure 5. Meanwhile, during the video, there is a moment when the CLC texture on the left side becomes dark. This corresponds to the intentional blocking of all light entering the CCD zoom lens in order to verify the influence of L3(light reflected from the zoom lens surface of the CCD system), as described in Fig. S3, on the spectrum displayed on the left side.

Video V2. Laser intensity spectrum and photonic bandgap (PBG) without filtering (600–620 nm)

Real-time spectrum of laser intensity and the photonic bandgap (PBG) in the 600–620 nm range, measured without a filter, corresponding to Fig. 6a′.

Video V3. Laser intensity spectrum without filtering (600–620 nm)

Real-time spectrum of laser intensity in the 600–620 nm range, measured without a filter, corresponding to Fig. 6b′.

Video V4. Laser intensity spectrum after F-LCLC filtering (600–620 nm)

Real-time spectrum of laser intensity in the 600–620 nm range, measured after passing through the **F-LCLC** filter, corresponding to Fig. 6c′.

Video V5. Laser intensity spectrum after F-RCLC filtering (600–620 nm)

Real-time spectrum of laser intensity in the 600–620 nm range, measured after passing through the **F-RCLC** filter, corresponding to Fig. 6d′.

Video V6. Laser intensity spectrum after F-88100 filtering (600–620 nm)

Real-time spectrum of laser intensity in the 600–620 nm range, measured after passing through the **F-88100** filter, corresponding to Fig. 6e′.

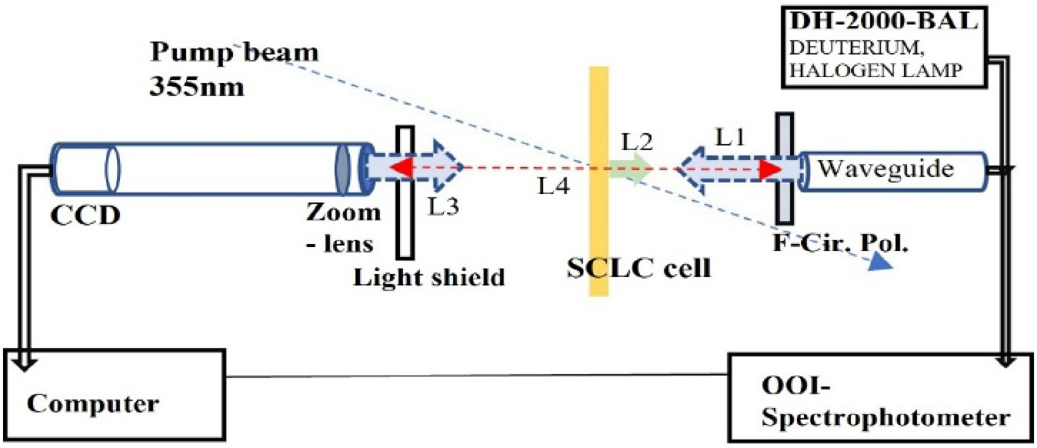

Fig. S3 Experimental setup to study PBG change and laser generation in the P-SCLC1 and the W-SCLC2 cells and the degree of circular polarization of the generated laser pulses: a red arrow is generated laser pulse from the SCLC cell, L1; white light from the deuterium and halogen lamps, L2; light reflected from the SCLC cell surface, L3; light reflected from the zoom lens surface of the CCD system, and L4(red arrow); generated laser pulses from the SCLC cell, F-Cir. Pol.; One of the circular polarizing plate filters of F-RCLC, F-LCLC, and F-88100; the pumping beam was focused on the SCLC cell surface using a lens (focal length, 20 cm) and irradiated to the SCLC cell surface with an incidence angle range between 15 and 40 degrees to obtain unusually strong absorption at that angle [R-S1]. At the same time, white light is irradiated in a direction perpendicular to the surface of CLC cells through the wave guide connected to the halogen and the deuterium lamps for measuring the PBG of the SCLC cell. After that, the laser (L4, red arrow in the figure) generated on the surface of the SCLC cell by the pump beam and the PBG spectral light reflected from the SCLC cell are transmitted to the OOI spectrophotometer through the same waveguide and then transmitted to the computer to be stored in the form of a spectrum. The laser pulses generated in the SCLC cell are emitted in both directions perpendicular to the SCLC cell surface, one towards the waveguide and the other towards the CCD. To determine the degree of circular polarization of the generated laser pulse, the three filters, F-88100, F-RCLC, and F-LCLC were placed one by one in front of the wave guide, so that the generated laser pulse passed through this filter and entered the wave guide. Since F-88100 is a filter that guarantees performance in low-intensity continuous wave laser light, the intensity of all pumping and generated laser pulses was used to be small intensity.

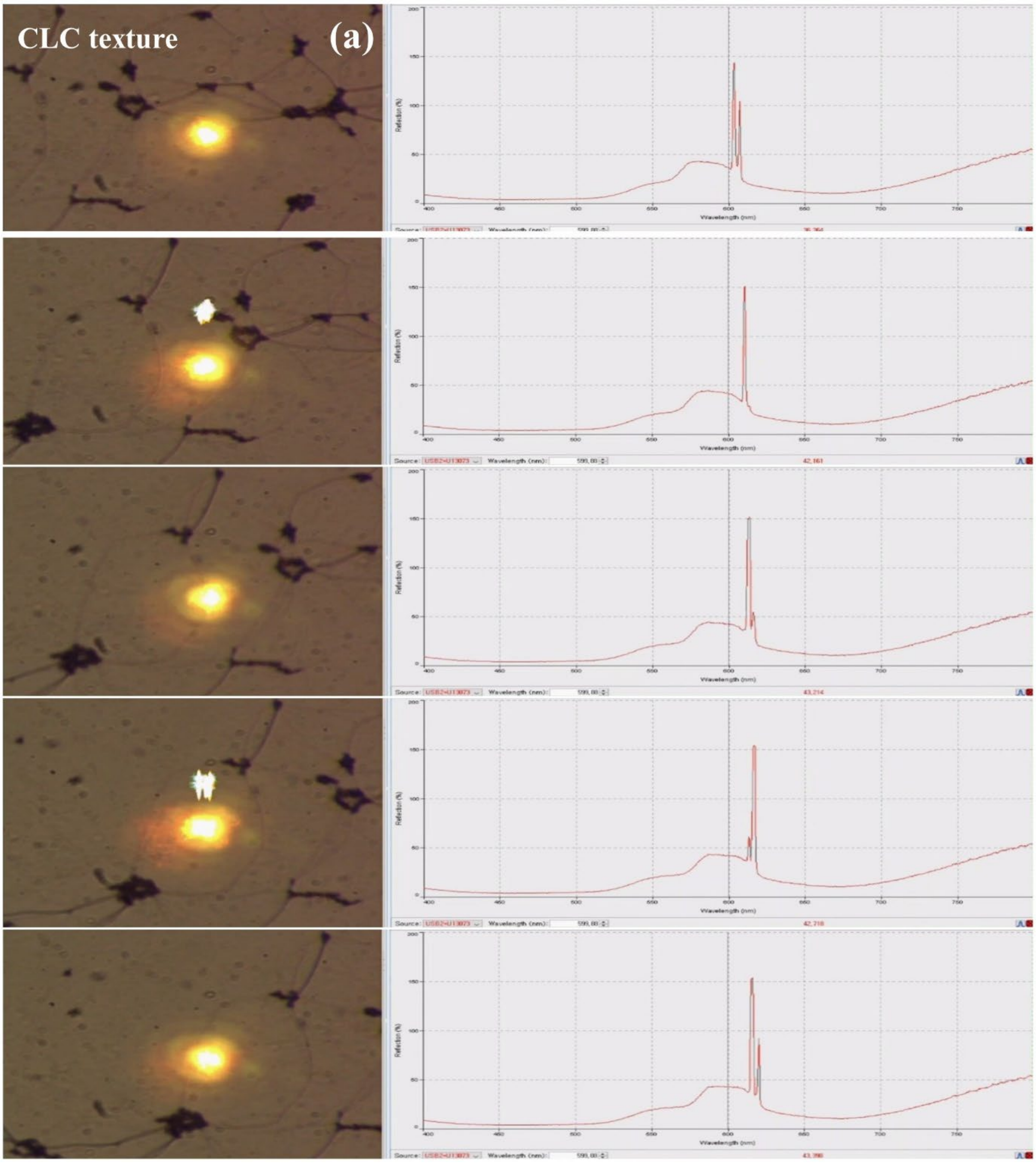

Fig. S4 (a) No-Filter + PBG: Five generated laser intensities and PBGs of the W-SCLC2 cell, within the range of 600 nm to 620 nm. The OOI lamp was turned on to check the SCLC cell texture and measure the location of PBG and the generated laser peak simultaneously. Mean value of 5 laser pulses is 118.6 within 600 nm to 620 nm.

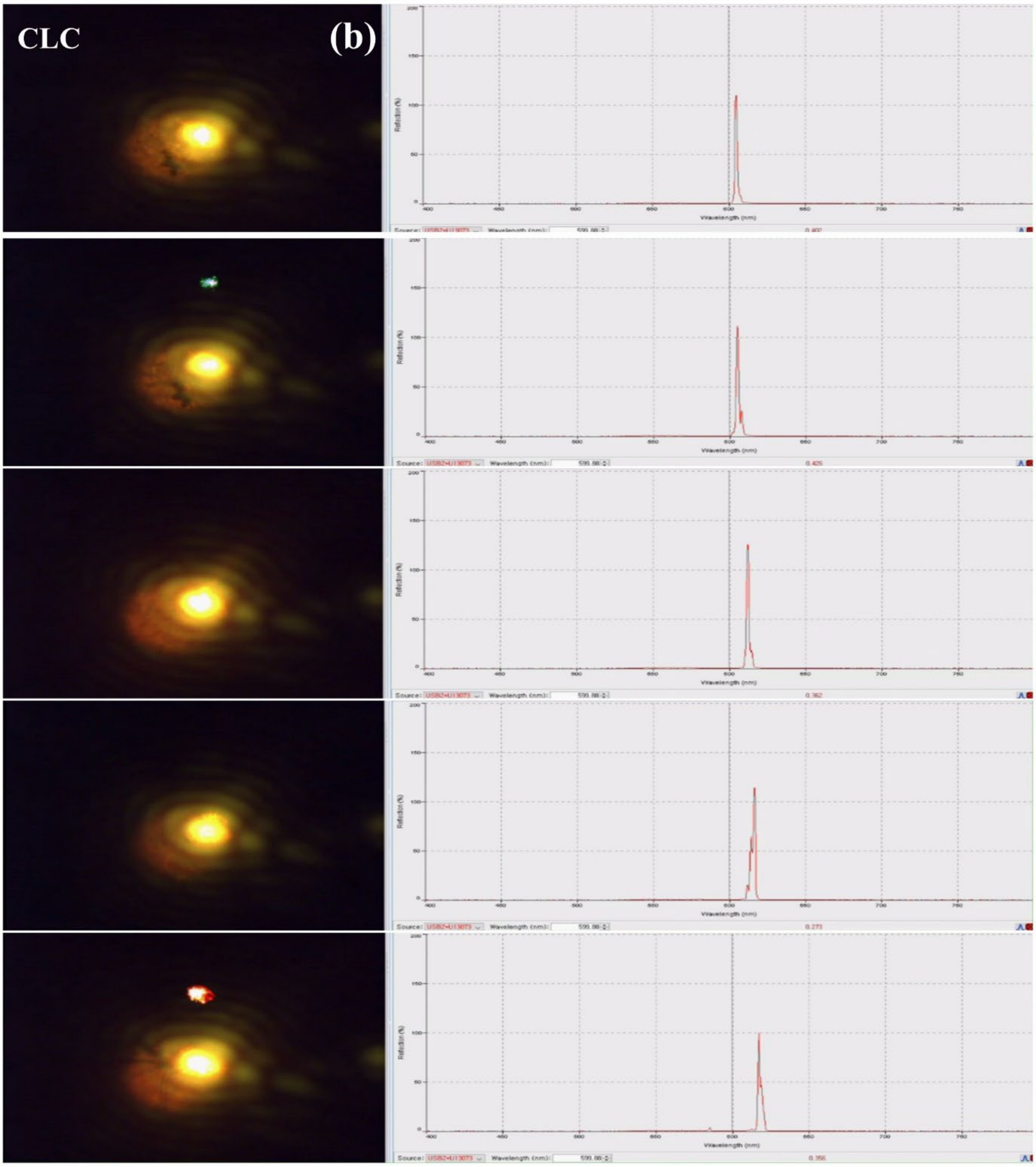

Fig. S4 (b) No-Filter: five generated laser intensities of the W-SCLC2 cell, within the range of 600 nm to 620 nm. The OOI lamp was turned off to measure only the generated laser peak intensity. Mean value of 5 laser pulses is 112.10 within 600 nm to 620 nm.

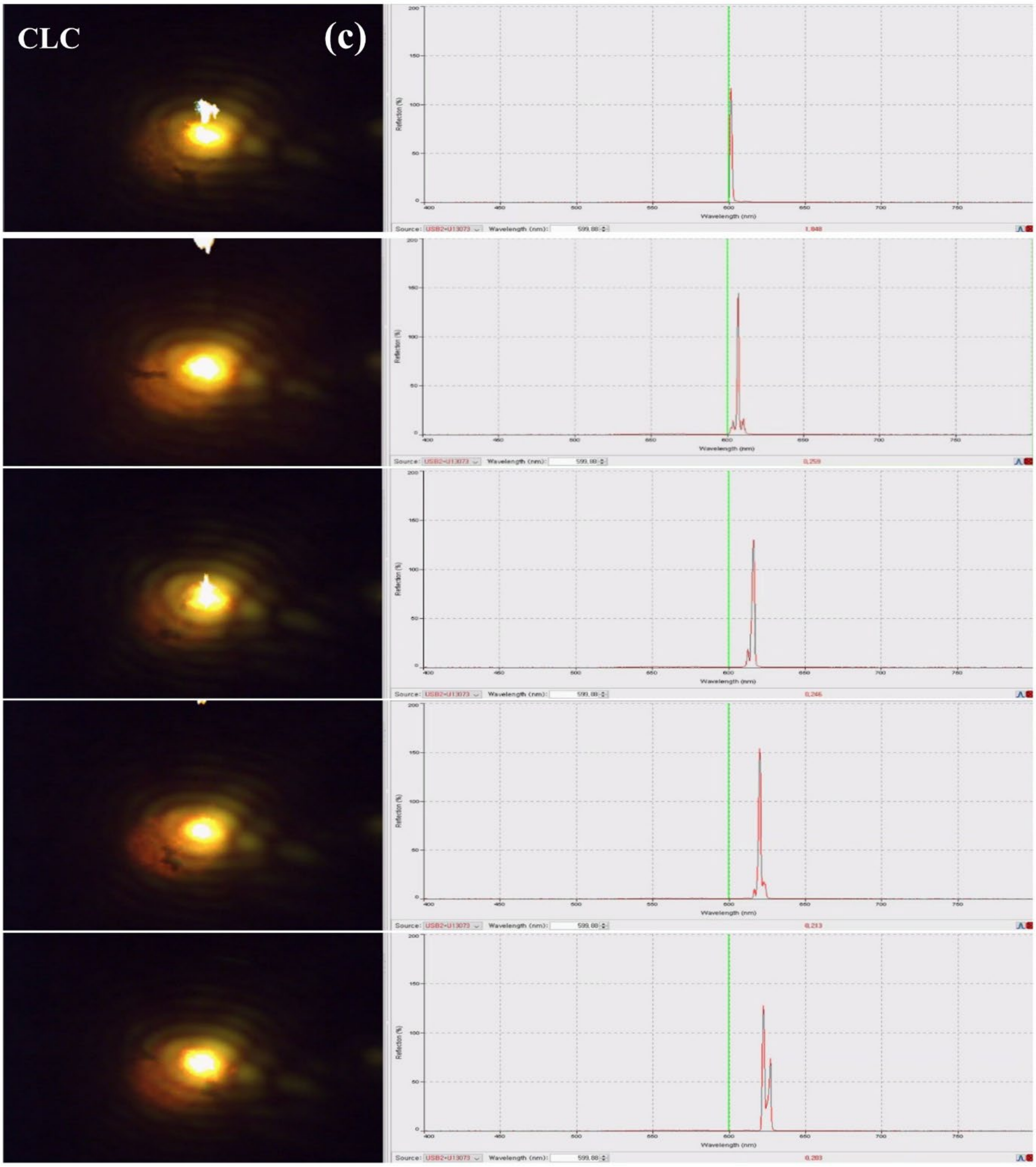

Fig. S4 (c) F-L-CLC613: five generated laser intensities of the W-SCLC2 cell after passing through the F-LCLC of the SCLC cell, within the range of 600 nm to 620 nm. The OOI lamp was turned off to measure only the generated laser peak intensity. Mean value of 5 laser pulses is 134.9 within 600 nm to 620 nm.

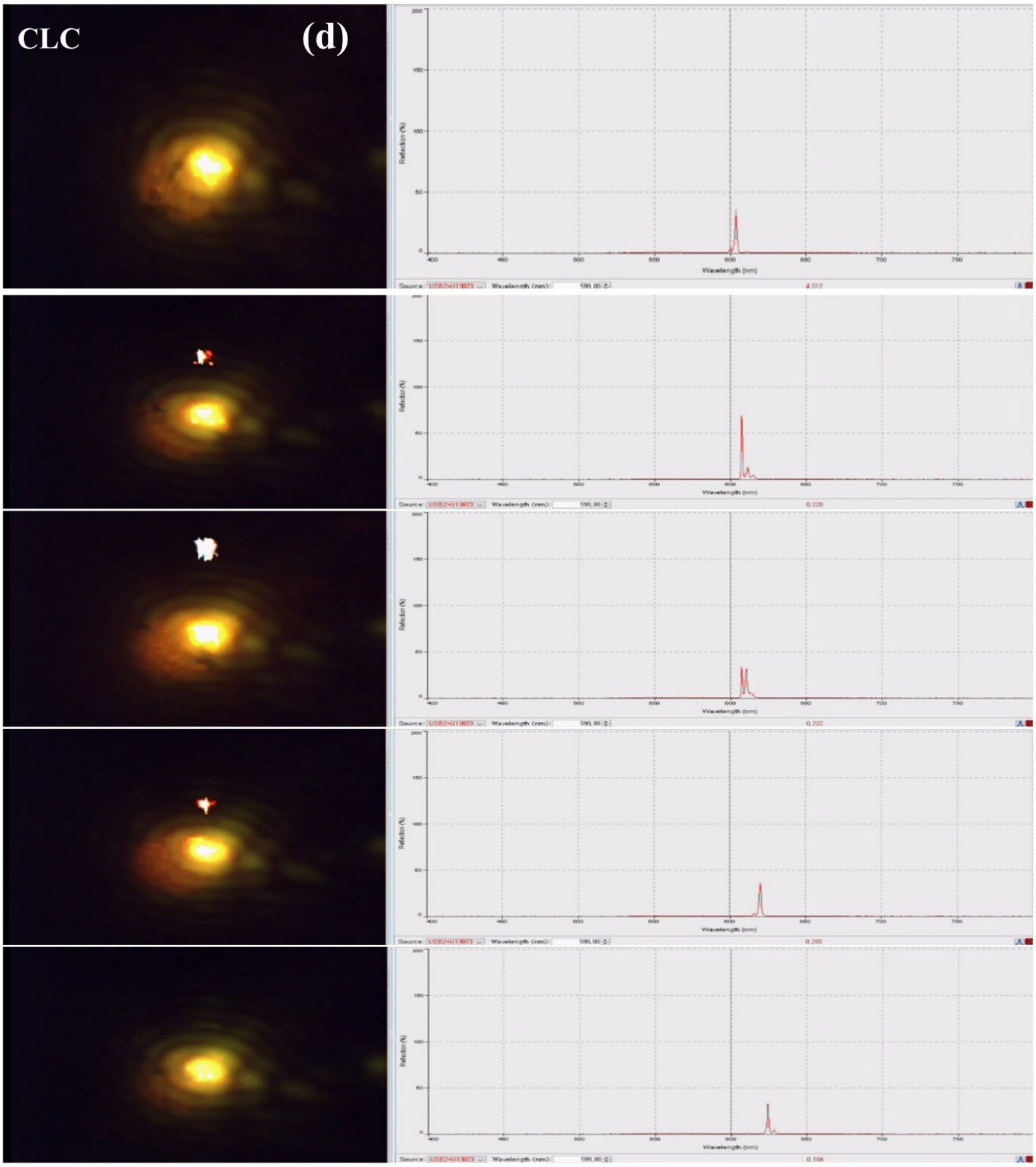

Fig. S4 (d) F-RCLC613: five generated laser intensities of the W-SCLC2 cell after passing through the F-RCLC of the SCLC cell, within the range of 600 nm to 620 nm. The OOI lamp was turned off to measure only the generated laser peak intensity. Mean value of 5 laser pulses is 41.10 within 600 nm to 620 nm.

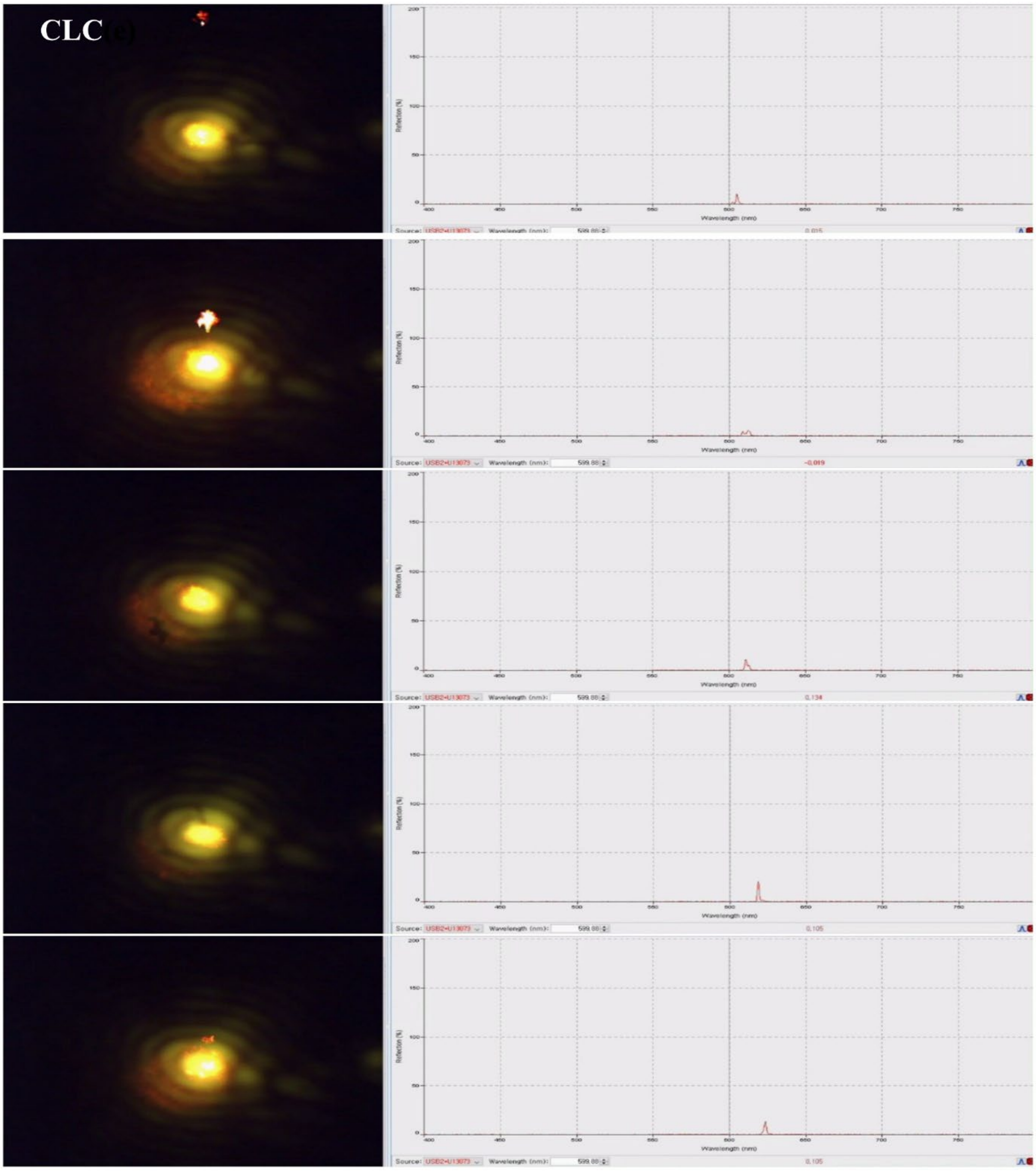

Fig. S4 (e) F-88100: five generated laser intensities of the W-SCLC2 cell after passing through the F-88100 of the SCLC cell, within the range of 600 nm to 620 nm. The OOI lamp was turned off to measure only the generated laser peak intensity. Mean value of 5 laser pulses is 12.02 within 600 nm to 620 nm.

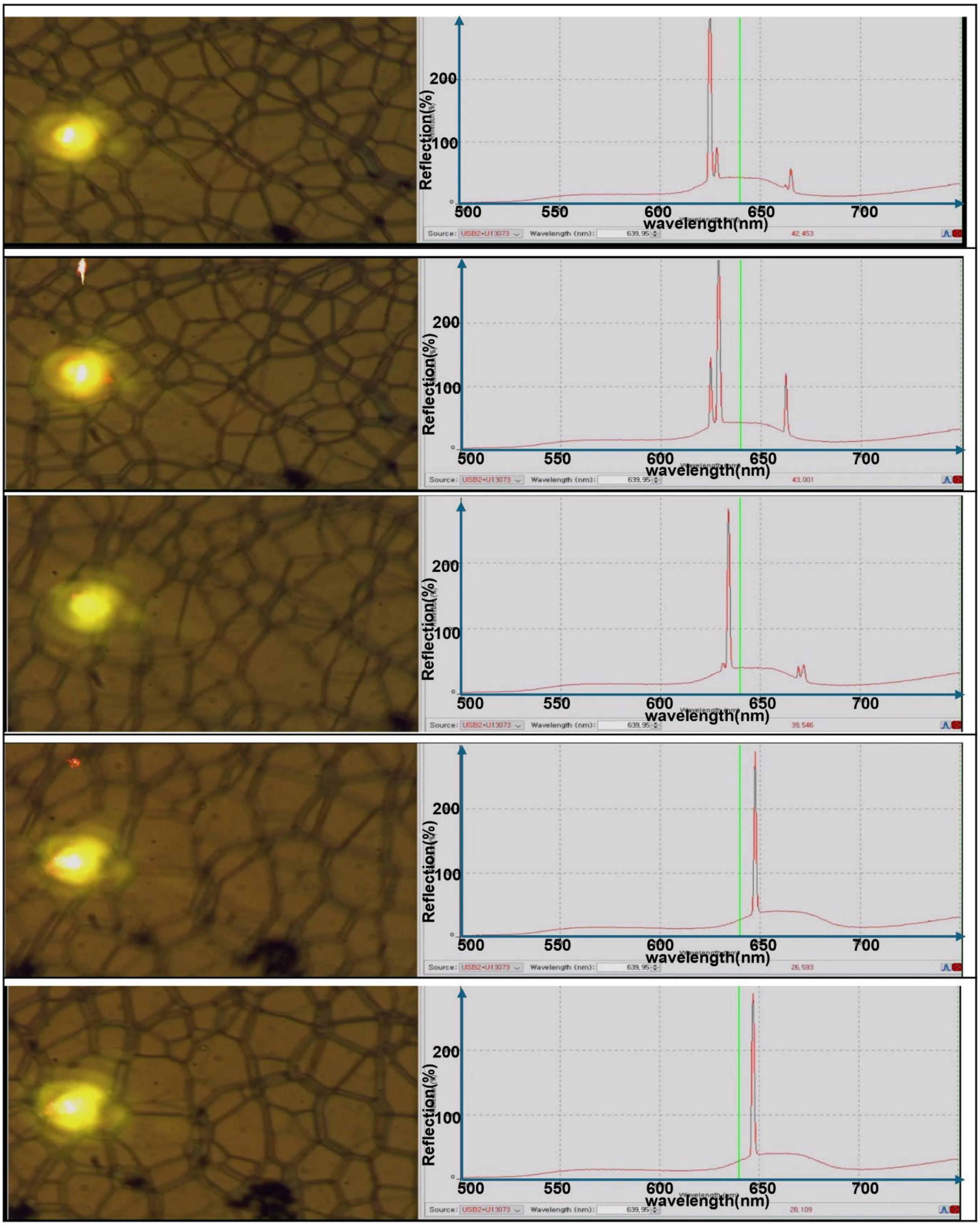

Fig. S4 (f) No-Filter + PBG: Five generated laser intensities and PBGs of the W-SCLC2 cell, within the range of 630 nm to 650 nm. The OOI lamp was turned on to check the SCLC cell texture and measure the location of PBG and the generated laser peak simultaneously. Mean value of 5 laser pulses is 255.4 within 630 nm to 650 nm.

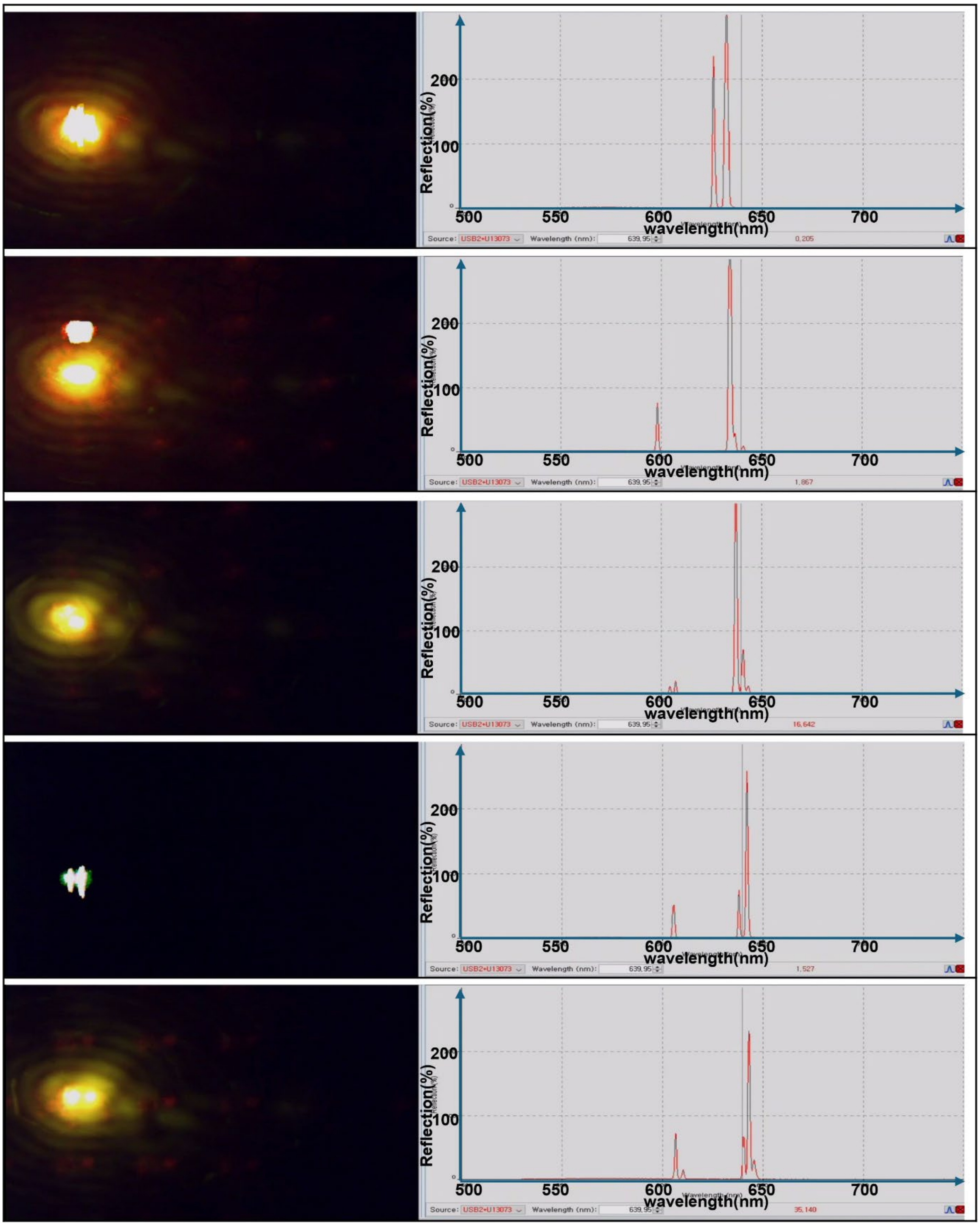

Fig. S4 (g) No-Filter: five generated laser intensities of the W-SCLC2 cell, within the range of 630 nm to 650 nm. The OOI lamp was turned off to measure only the generated laser peak intensity. Mean value of 5 laser pulses is 278.6 within 630 nm to 650 nm.

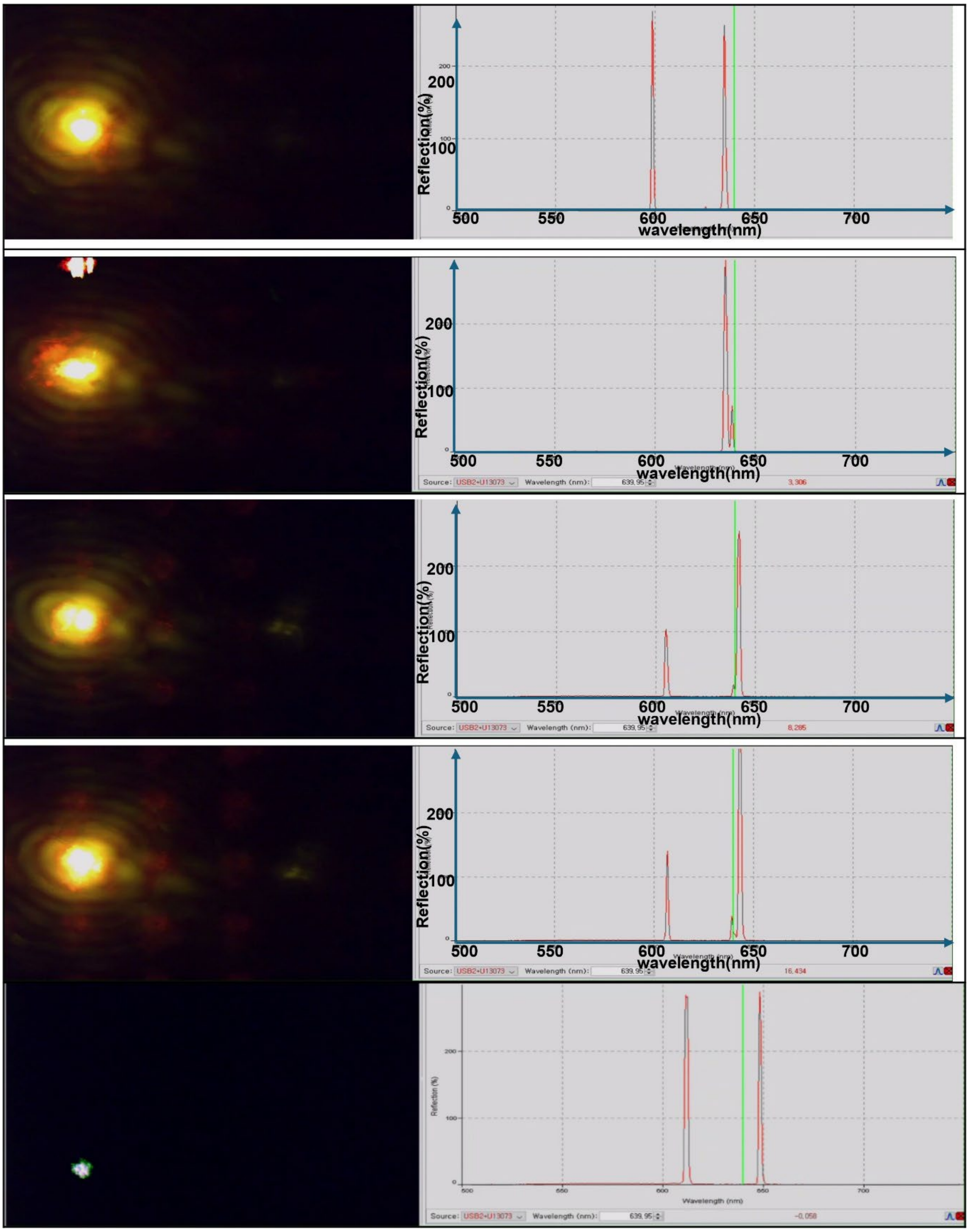

Fig. S4 (h) F-LCLC648: five generated laser intensities of the W-SCLC2cell after passing through the F-88100 of the SCLC cell, within the range of 630 nm to 650 nm. The OOI lamp was turned off to measure only the generated laser peak intensity. Mean value of 5 laser pulses is 280.80 within 630 nm to 650 nm.

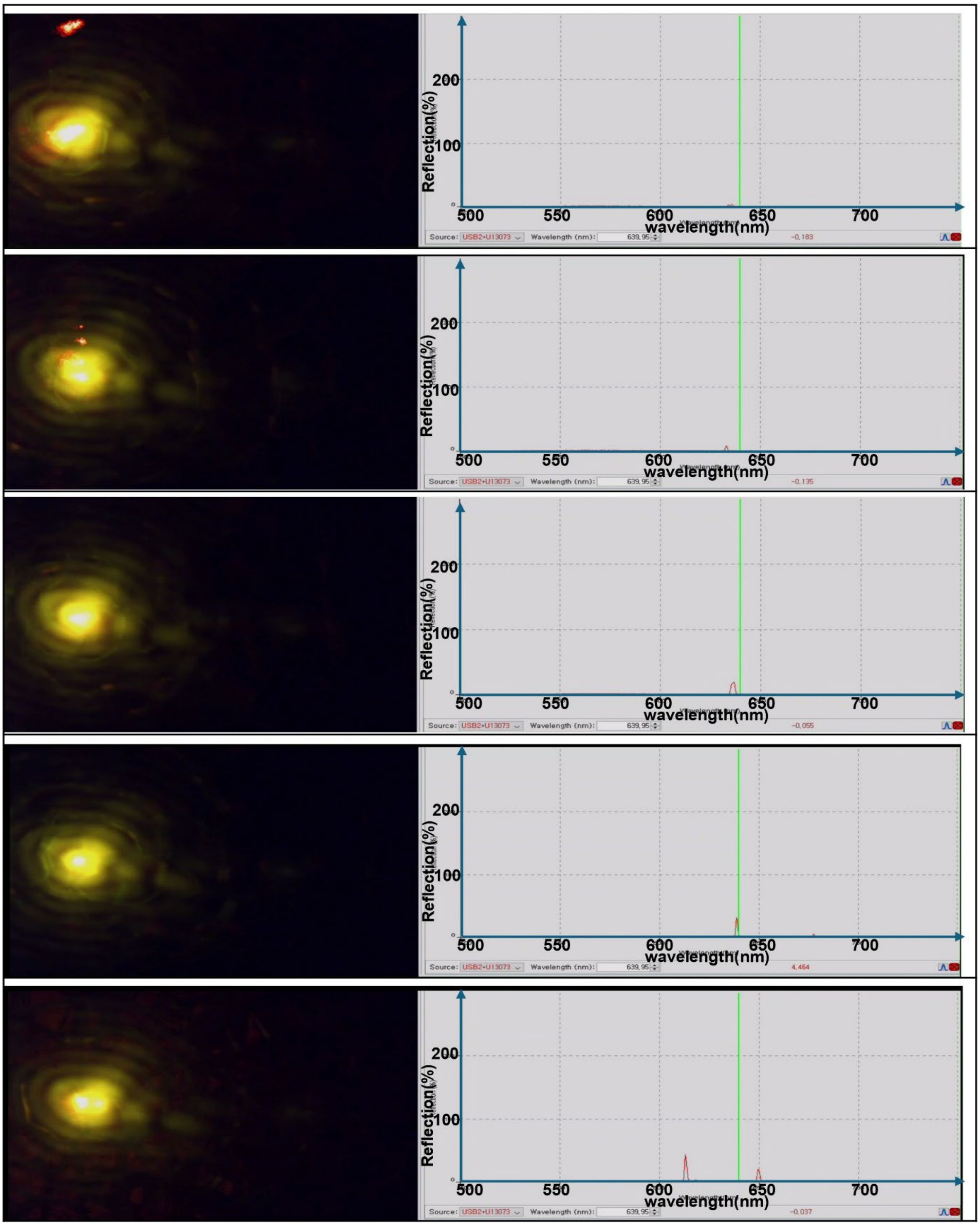

Fig. S4 (i) F-RCLC648: five generated laser intensities of the W-SCLC2cell after passing through the F-RCLC648 of the SCLC cell, within the range of 630 nm to 650 nm. The OOI lamp was turned off to measure only the generated laser peak intensity. Mean value of 5 laser pulses is 16.00 within 630 nm to 650 nm.

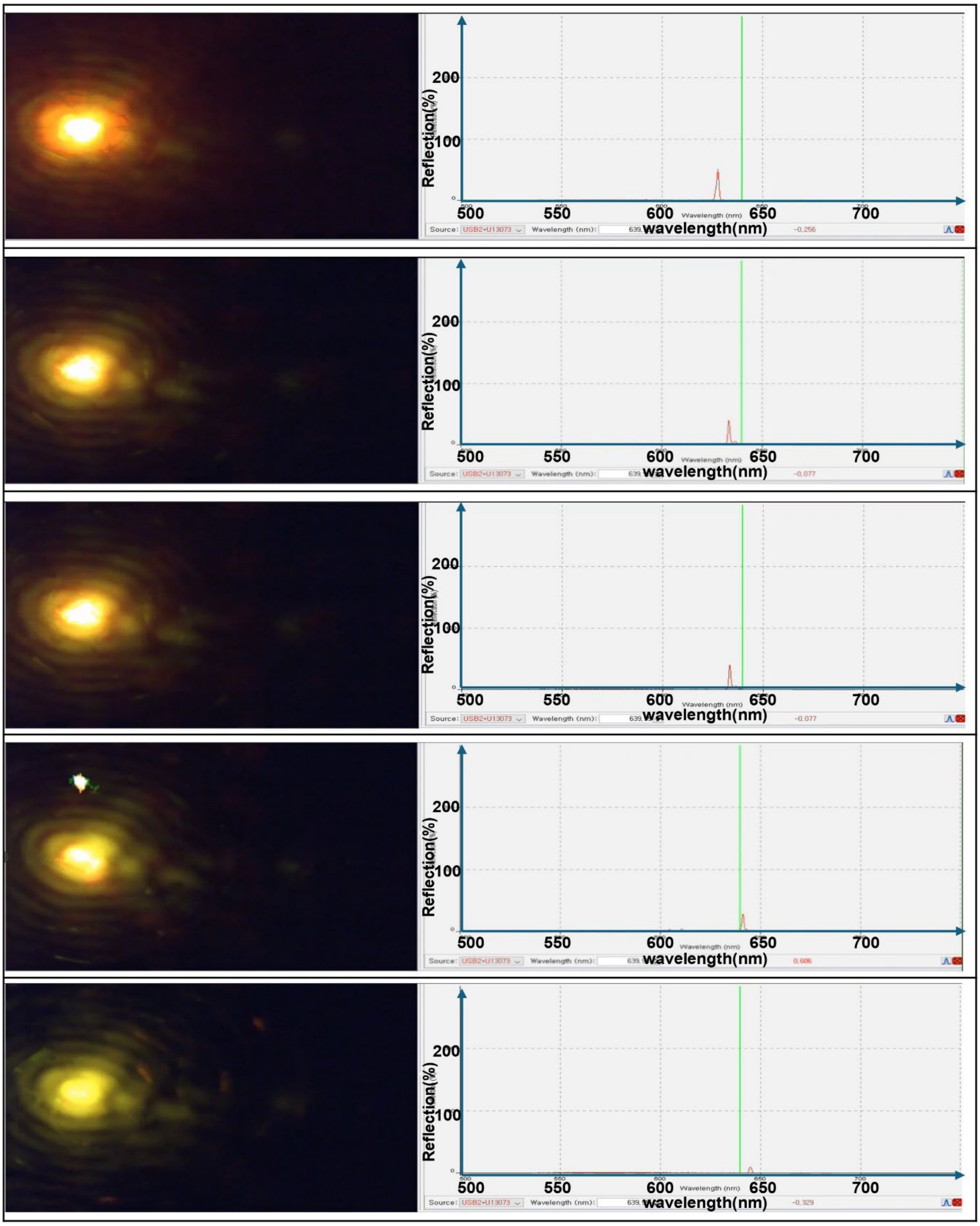

Fig. S4 (j) F-88100: five generated laser intensities of the W-SCLC2 cell after passing through the F-88100 of the SCLC cell, within the range of 630 nm to 650 nm.The OOI lamp was turned off to measure only the generated laser peak intensity. Mean value of 5 laser pulses is 34.00 within 630 nm to 650 nm.

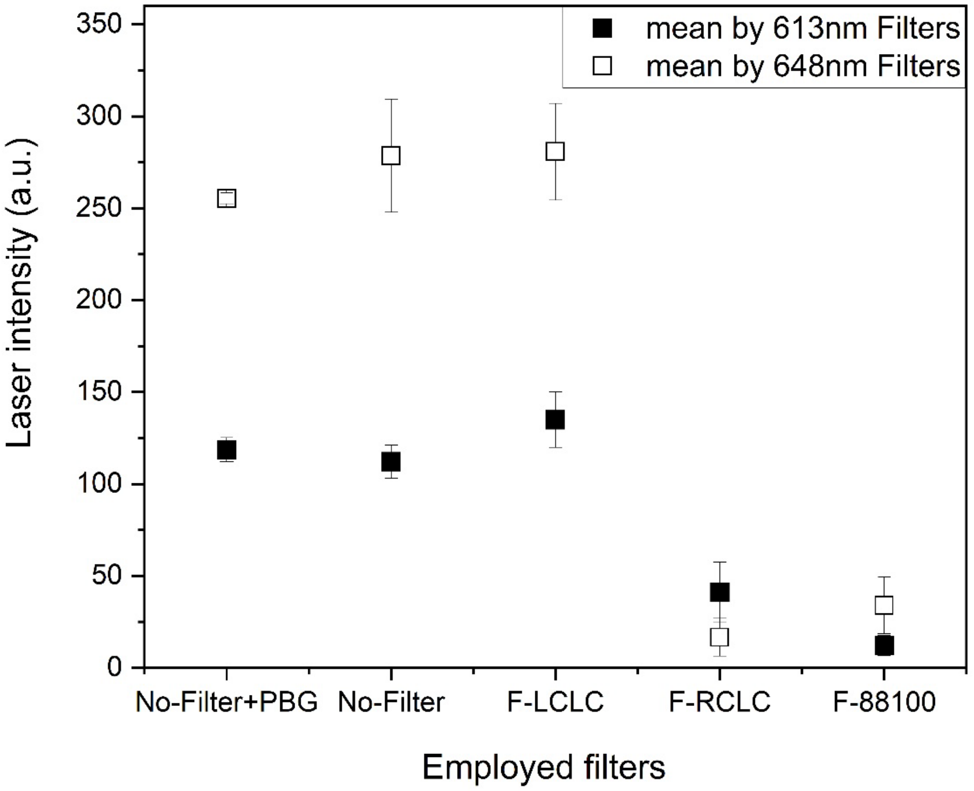

Fig. S5 Average laser intensities of five laser peaks (600–620 nm (■) and 630nm-650 nm (□)) generated from the W-SCLC2 cell under 5 different filter conditions in the Figure S4 (a,b,c,d,e; by 613nm filters) and (f, g, h, i, j; by 668nm filters); No-Filter + PBG (the laser intensity measured together with the photonic bandgap without any filter through the waveguide), No-Filter (the laser intensity measured without any filter), and the intensities after passing through F-LCLC613, F-RCLC613, F-LCLC648, F-RCLC648, and F-88100, respectively.

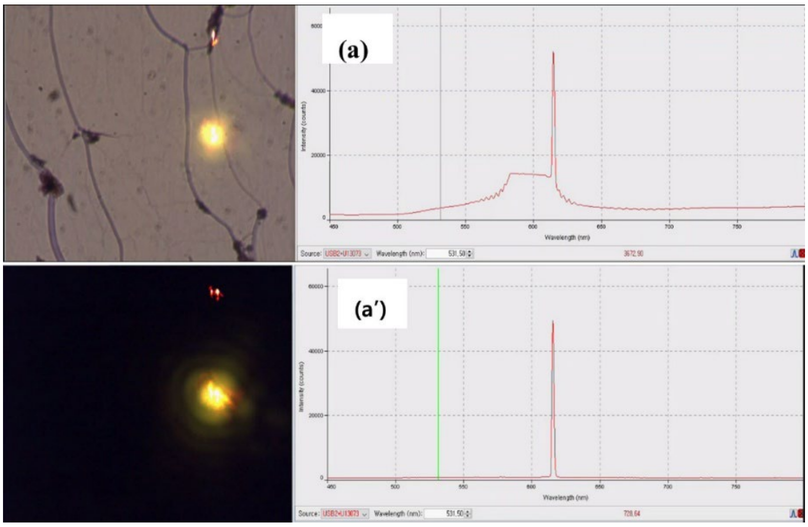

Fig. S6 (a, a’) No-Filter + PBG: a generated laser peak intensity and PBG of the P-SCLC1 cell, within the range of 600 nm to 620 nm. The OOI lamp was turned on (a) and off (a’) to check the SCLC cell texture and measure the location of PBG and the generated laser peak simultaneously.

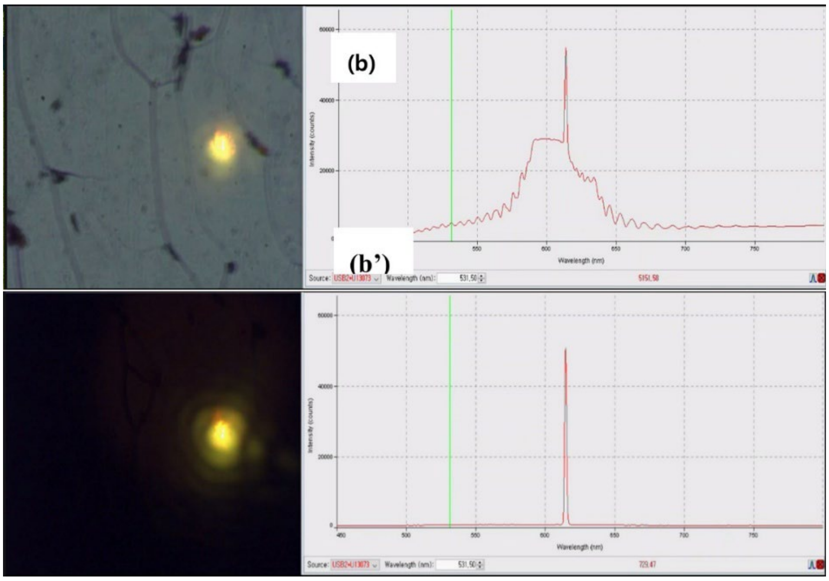

Fig. S6 (b, b’) F-LCLC: a generated laser intensity of the P-SCLC1 cell after passing through the F-LCLC cell, within the range of 600 nm to 620 nm. The OOI lamp was turned on (a) and off (a’) to check only the generated laser peak intensity.

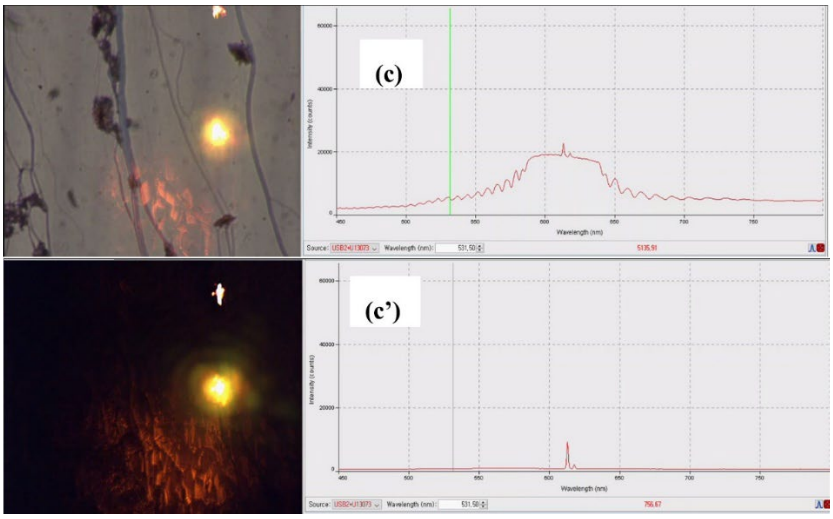

Fig. S6 (c, c') F-RCLC: a generated laser intensity of the P-SCLC1 cell after passing through the F-RCLC cell(c), within the range of 600 nm to 620 nm. The OOI lamp was turned off to measure only the generated laser peak intensity(c').

S-P1: Proof of eq. (2)

$$I = \frac{1}{2}\ [(\tau_L + \tau_R)S_0 + (\tau_L - \tau_R)S_3] = \tau_L I_L + \tau_R I_R \qquad (2)$$

Here, S_0 is the total intensity of light, ie., S_0=(I_L + I_R).

Here, I_L is the intensity of LCP component, and I_R is the intensity of RCP component. S_3 is the Stoke's parameter of circularly polarized light, ie., S_3=($I_L - I_R$). $S_3 > 0$ implies left polarization dominates; and $S_3 < 0$ implies right polarization dominates. τ_L is transmittance of a filter for LCP and τ_R is transmittance of a filter for RCP. So, practical intensity through a filter can be expressed by $I = \tau_L I_L + \tau_R I_R$. Here, $I_L = \frac{S_0 + S_3}{2}$, $I_R = \frac{S_0 - S_3}{2}$.

So, $I = \tau_L \frac{S_0 + S_3}{2} + \tau_R \frac{S_0 - S_3}{2}$. It makes the same equation as (2):

$I = \frac{1}{2}\ [(\tau_L + \tau_R)S_0 + (\tau_L - \tau_R)S_3]$.

S-P2: Proof of eq.,

$$I_{LCLC} + I_{RCLC} = (A + B) \cdot (I_L + I_R) = 1.04 \cdot S_0$$

Here, we put $A = \tau_H$, and $B = \tau_L$. τ_H is transmittance of preferred helicity and τ_L is transmittance of the opposite helicity.

Using average transmittance of non-polar incident light is 0.52(Table-S1), $\frac{A+B}{2} = T_{UP} = 0.52 \Rightarrow A + B = 1.04$. So, the intensity of LCLC + RCLC equals to $(A + B)(I_L + I_R)$ and $(I_L + I_R) = S_0$. That is, the intensity from both filters is proportional to S_0.

S-P3: Proof of eq. (4):

$$I_{LCLC} = A \cdot I_R + B \cdot I_L,$$

$$I_{RCLC} = A \cdot I_L + B \cdot I_R.$$

The LCLC filter is designed to transmit RCP and reflect LCP. So, most of right circular component transmits as $A = \tau_H$. But some portion of left circular component leaks through the filter. So, left circular component transmits as $B = \tau_L$(leak component). So, $I_{LCLC} = A \cdot I_R + B \cdot I_L$. On the other hand, the RCLC filter is designed to transmit LCP while reflecting RCP. So, $I_{RCLC} = A \cdot I_L + B \cdot I_R$.

Table-S1. Experimental data values of transmittance to unpolarized incident light (Figure 2(a)) and average intensity of five laser pulses passing through each of the three filters (Figure S5). The † indicates a laser pulse measured within a range of 600 to 620 nm.

Quantity	Value
Laser intensity of incident, S_0	169.23 (converted)
I_{LCLC} -Laser intensity after passing through F-LCLC613	134.90† (ref. Fig. S5)
I_{RCLC}-Laser intensity after passing through F-RCLC613	41.10† (ref. Fig. S5)
I_{88100}-Laser intensity after passing through F-88100	12.02† (ref. Fig. S5)
Unpol. light trans, of F-LCLC613	52% (ref. Fig. 2(a))
Unpol. light trans. of F-RCLC613	52% (ref. Fig. 2(a))
Unpol. light trans. of F-88100	38.68% (ref. Fig. 2(a))

Table-S2. Experimental data values of transmittance to unpolarized incident light (Figure 2(b)) and average intensity of five laser pulses passing through each of the three filters (Figure S5). The † indicates a laser pulse measured within a range of 630 to 650 nm.

Quantity	Value
Laser intensity of incident, S_0	294.51 (converted)
I_{LCLC} -Laser intensity after passing through F-LCLC648	280.80† (ref. Fig. S5)
I_{RCLC}-Laser intensity after passing through F-RCLC648	16.66† (ref. Fig. S5)
I_{88100}-Laser intensity after passing through F-88100	34† (ref. Fig. S5)
Unpol. light trans, of of F-LCLC648	51% (ref. Fig. 2(b))
Unpol. light trans. of F-RCLC648	50% (ref. Fig. 2(b))
Unpol. light trans. of F-88100	38.68% (ref. Fig. 2(a))

S-Cal: calculation g in the 630nm~650nm range:

For F-LCLC648 and F-RCLC648, define $A \equiv \tau_{pref}$ (transmission of the preferred helicity) $and\ B \equiv \tau_{leak}$ (transmission of the opposite helicity), Then $A + B = 2 \cdot T_{UP} = 1.01$ (since $T_{LCLC} = 51\%$, T_{RCLC} =50%, see Fig. 2 (or Table S2).

Because real analyzers are imperfect, A ≤1 and B >0.

F-LCLC preferentially transmits right circular polarized light (leaking left polarized light) and F-RCLC preferentially transmits left polarized light (leaking right circular polarized light).

When I_{LCLC} is laser intensity after passing through F-LCLC648 and , I_{RCLC} is laser intensity after passing through F-RCLC648, hence [S-P**2**, S-P3]

$$I_{LCLC} = A \cdot I_R + B \cdot I_L, \quad (3)$$

$$I_{RCLC} = A \cdot I_L + B \cdot I_R$$

(4)

Summing eq. (3) and (4) gives $I_{LCLC} + I_{RCLC} = (A + B) \cdot (I_L + I_R) = 1.01 \cdot S_0$,

Using the measured values (Table S1 and Table S2) $I_{LCLC} =$ 280.8 and $I_{RCLC}=$ 16.66,

$$S_0 = (I_{LCLC} + I_{RCLC})/(A + B) = \frac{280.8+16.66}{1.01} = 294.51 \quad (5)$$

Taking difference,

$$I_{LCLC} - I_{RCLC} = -(A - B) \cdot (I_L - I_R) = -(A - B)S_3 \quad (6)$$

For the commercial circular polarizer (F-88100), let $t_L\ and\ t_R$ denote its left /right circular

polarization transmission coefficients. With unpolarized transmittance $T_{UP,88100} = 0.3868$, We have $t_L + t_R = 2 \cdot T_{UP,88100} = 0.7736$. The measured transmitted intensity is $I_{88100} = 34$, so

$$I_{88100} = \frac{1}{2}\left[(t_L + t_R)S_0 + (t_L - t_R)S_3\right]$$

$$2\,I_{88100} - (t_L + t_R)S_0 = (t_L - t_R)(I_L - I_R) \qquad (7)$$

By combination of (6) and (7),

$$\frac{(t_L - t_R)}{\tau_{pref} - \tau_{leak}} = \frac{(t_L + t_R)S_0 - 2\,I_{88100}}{I_{LCLC} - I_{RCLC}}$$

$$= (0.7736 * 294.51 - 2 * 34)/(280.8 - 16.66) \approx 0.6051 \qquad (8)$$

With $0 \le t_{L,R} \le 1$, $|t_L - t_R| \le 0.7736$. Therefore,

$$|A - B| \le \frac{|t_L - t_R|}{1.13984} \le \frac{0.7736}{0.6051} \approx 1.278 \qquad (9)$$

Finally, $g = \frac{2(I_L - I_R)}{S_0} = \frac{2|I_{LCLC} - I_{RCLC}|}{S_0|\tau_{pref} - \tau_{leak}|} \ge \frac{2 \times 264.14}{294.51 \times 1.278} \approx 1.4$,

where $264.14 = 280.8 - 16.66$

Additional measurements performed at lower pump power (reducing saturation effects) exhibited the same polarization-selective contrast trend, confirming that the high g values reported here are not artefacts of saturation. These auxiliary data are available upon request.

Reference

[R-S1] J. Schmidtke, W. Stille, and H. Finkelmann, "Defect Mode Emission of a Dye Doped Cholesteric Polymer Network," Phys. Rev. Lett. **90**, 083902 (2003).
